# Conditional Latent Diffusion for Synthetic Brain MRI in Alzheimer’s Disease: A Preprocessing-Focused Pipeline

**DOI:** 10.3390/jimaging12090426

**Published:** 2026-09-09

**Authors:** Soheil Fallah, Nitsa J. Herzog

**Affiliations:** Department of Computer and Information Sciences, Northumbria University London, London E1 7HT, UK; fallah.soheil@northumbria.ac.uk

**Keywords:** brain MRI synthesis, latent diffusion models, Alzheimer’s disease, medical image preprocessing, synthetic data generation, small-cohort learning, generative model memorisation, classifier-free guidance, ADNI

## Abstract

Deep learning for Alzheimer’s disease (AD) detection from structural magnetic resonance imaging (MRI) needs large, labelled datasets, yet many cohorts hold only a few hundred participants, for which conventional augmentation adds little anatomical diversity. In a two-stage pipeline, a variational autoencoder compressed 256 × 256 coronal slices to a 32 × 32 × 8 latent space, and a class-conditional latent diffusion model under classifier-free guidance generated AD and cognitively normal (CN) images using 295 participants from the Alzheimer’s Disease Neuroimaging Initiative (ADNI). The pipeline reached a Kernel Inception Distance (KID) of 0.030 ± 0.002 and a bias-corrected Fréchet Inception Distance (FID_∞_) of 43.82. A controlled ablation varying preprocessing alone improved KID by 0.0147 and precision by 0.069, both with 95% intervals excluding zero. FID did not separate the configurations. A ResNet-18 trained only on synthetic slices and tested on 44 held-out real participants (18 AD, 26 CN), each scored as the mean probability over twenty slices, reached an area under the curve of 0.779 ± 0.031 against 0.869 ± 0.027 for real data; the difference was not distinguishable at this sample size. No instance memorisation was found among 880 samples, and a size-matched control exposed a 27.7-percentage-point inflation in the standard memorisation metric. Preprocessing, therefore, measurably affects synthesis quality at the small-cohort scale, though not on every measure.

## 1. Introduction

Alzheimer’s disease is the most common cause of dementia, affecting over 55 million people worldwide, with projections reaching 139 million by 2050 [1]. The disease follows a progressive neurodegenerative course in which neurofibrillary tangle pathology begins in the entorhinal cortex, spreads to the hippocampus, and eventually reaches neocortical association areas [2]. Structural brain magnetic resonance imaging (MRI) can detect the atrophy years before clinical diagnosis, making hippocampal volume loss, ventricular enlargement, and cortical thinning established imaging biomarkers for early detection.

Deep learning has been widely applied to the automated detection of Alzheimer’s disease from structural MRI. A systematic review of 116 studies reported a mean Convolutional Neural Network (CNN) accuracy of 78.5% for predicting conversion from mild cognitive impairment (MCI) to Alzheimer’s disease, with 107 of the 116 studies relying on data from the ADNI database [3]. Anatomically informed feature design can exceed this average, as Herzog and Magoulas [4] separated cognitively normal subjects from Alzheimer’s disease cases at 93.0% accuracy on ADNI data using machine learning applied to features of brain asymmetry. These methods require large, labelled datasets to train effectively, yet many clinical research groups have access to cohorts of only 50 to 500 subjects. Class imbalance compounds the problem, with cognitively normal participants typically outnumbering those with Alzheimer’s disease. Multi-site acquisition introduces further confounds, as scanner-dependent intensity distributions across ADNI’s 54 sites are classifiable by manufacturer with approximately 99% accuracy [5]. Privacy regulations under frameworks such as the UK GDPR additionally constrain the sharing of medical imaging data between institutions, even when de-identified.

Generative models offer a fundamentally different approach to data scarcity. Rather than augmenting existing images through geometric transformations, which cannot introduce new anatomical variation, generative models learn the underlying data distribution and synthesise new samples from it [6]. Generative adversarial networks produce sharp outputs but suffer from mode collapse and training instability that worsens as the dataset size decreases [7]. Denoising diffusion probabilistic models avoid these instabilities through iterative noise prediction [8], and latent diffusion models (LDMs) brought this approach to practical resolution by performing the diffusion process in a compressed latent space rather than at pixel resolution, reducing computational cost by 16- to 64-fold [9].

Several studies have applied LDMs to brain MRI. Pinaya et al. [10] trained a VQ-VAE autoencoder paired with a conditional diffusion model on 31,740 UK Biobank T1-weighted volumes at 160 × 224 × 160 voxels. Ventricular volume conditioning yielded a correlation of *r* = 0.972 between the specified and measured volumes. The UK Biobank cohort is predominantly healthy, though, and the study did not condition on disease status. Müller-Franzes et al. [11] introduced Medfusion, a latent DDPM with eightfold spatial compression, and demonstrated generalisation across fundoscopy, histopathology, and chest radiography, achieving FID values of 11.63 to 30.03 and consistently outperforming GAN baselines in both precision and recall. Khader et al. [12] extended this to 3D volumes using VQ-GAN with DDPM across chest CT, brain MRI, and knee MRI. In a radiologist assessment, 50 of 50 generated ADNI brain MRIs were rated at least largely realistic. Dhinagar et al. [13] trained a conditional LDM on 4098 ADNI T1-weighted scans from 1188 subjects and showed a 3-percentage-point improvement in AD classification AUC when augmenting with synthetic data, using classifier-free guidance with 15% label dropout and a guidance scale of 2. Counterfactual heat maps from that study highlighted ventricular and temporal regions consistent with known atrophy patterns. Across these studies, two characteristics recur. All operate at cohort scales of 998 to 31,740 subjects, and the preprocessing methodology is minimally documented. Dhinagar et al. describe their preprocessing (N4 bias correction, skull stripping, linear registration, and min–max scaling) in a single paragraph. Pinaya et al. devote a brief subsection to their UK Biobank pipeline. Müller-Franzes et al. provide no brain MRI preprocessing details at all.

This gap matters because preprocessing decisions directly determine what the generative model receives as input. Without spatial registration, anatomical structures occupy different voxel positions across subjects, forcing the diffusion model to average over misaligned features and producing blurred outputs. Without principled slice selection, coronal sections may sample diagnostically uninformative regions while under-representing the hippocampus and entorhinal cortex, where Alzheimer’s pathology is most pronounced and where classification evidence is strongest. Mendoza-Léon et al. [14] classified AD at 90.0% accuracy from single coronal slices, and Chen et al. [15] improved diagnosis using a slice-level attention mechanism that emphasises informative slices and discards redundant ones. Despite this, no published work documents preprocessing at this level of detail for brain MRI synthesis or reports a same-cohort comparison between a baseline and an anatomically informed pipeline at a small-cohort scale. Variable-density anatomical slice selection, which densely samples disease-relevant regions while sparsely covering reference anatomy, has not been combined with generative modelling in the synthesis literature.

This study addresses these gaps through a two-stage conditional LDM pipeline for generating synthetic 2D coronal brain MRI conditioned on Alzheimer’s disease diagnosis, trained on 295 subjects from the ADNI. The pipeline comprises a VAEGAN autoencoder [16] that compresses 256 × 256 images into a 32 × 32 × 8 latent space, followed by a conditional LDM with classifier-free guidance. Two pipeline configurations were applied to the same 295 subjects. The baseline (v1) omitted spatial registration, selected slices by relative position within each subject’s own brain extent, and encoded into a four-channel latent, with a differently weighted LPIPS (Learned Perceptual Image Patch Similarity) [17] term. The enhanced version (v2) incorporated MNI152 registration, variable-density anatomical slicing informed by Braak staging neuropathology, an eight-channel latent at 256 × 256, and a literature-informed LPIPS weight of 0.5. Because these changes were applied together, that comparison shows the combined effect of the redesigned pipeline; a controlled ablation, in which only the preprocessing of the input data varied, isolates the contribution of preprocessing itself.

Thus, the work makes four contributions. First, the six-stage preprocessing pipeline with MNI152 registration and anatomically guided slice selection is documented in detail not found in existing brain MRI synthesis literature, and a controlled ablation, in which the enhanced architecture was trained on the baseline data so that only the preprocessing varied, improves KID by 0.015 (95% CI −0.023 to −0.008) and precision by 0.069 (0.004 to 0.123). Meanwhile, the FID interval spans zero. Second, a classifier trained on synthetic images reaches a subject-level AUC of 0.779 ± 0.031 on real test data across ten training seeds, from a cohort a fraction of the size used by the nearest comparable study [13]. Third, a 27.7-percentage-point set-size confound in the Scardace et al. [18] nearest-neighbour memorisation metric is identified and quantified, with a corrected protocol using equal-sized reference sets and real-image control baselines under which no near-copy instances were identified across 880 generated samples. Fourth, a multi-tier evaluation protocol combining bias-corrected FID_∞_ [19], KID as primary distributional metric [20], subject-level TSTR with the Yagis et al. [21] correction, and memorisation severity grading provides a practical framework for honest assessment at a small-cohort scale.

The rest of this paper is organised as follows. Section 2 describes the dataset, preprocessing pipeline, model architecture, training protocol, and evaluation framework. Section 3 presents the experimental results and compares model performance. Section 4 discusses the findings in the context of existing literature and identifies limitations. Section 5 concludes with a summary of contributions and directions for future work.

## 2. Materials and Methods

### 2.1. Dataset

**Figure 1:**
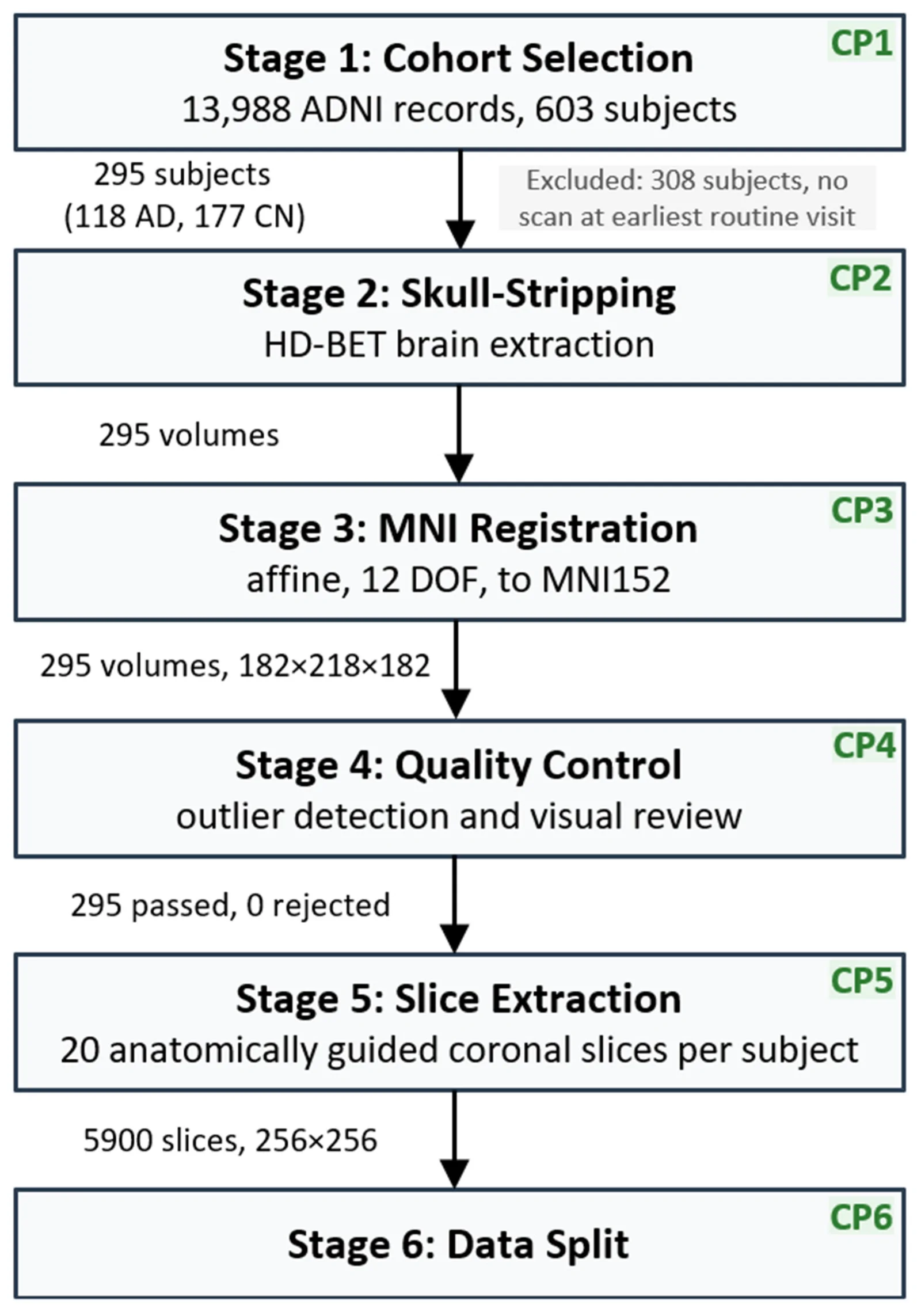
Six-stage preprocessing pipeline, from ADNI query to model-ready coronal slices. Grey boxes denote processing stages; green tags denote the checkpoint gate that closes each stage. The gates verify, in order: one volume per subject with preprocessing priority confirmed (CP1); valid NIfTI output (CP2); correct registered shape with ventricles aligned (CP3); a rejection rate below 5% (CP4); correct slice resolution and consistent anatomy (CP5); and no subject leakage across partitions (CP6). Stage 6 divides the 5900 slices into 4080 training, 940 validation and 880 test slices, stratified jointly on diagnosis and acquisition site. Of the 295 selected volumes, 83.4% carry the highest-priority combination of intensity and distortion corrections.

Data were obtained from the Alzheimer’s Disease Neuroimaging Initiative [22], a multi-site longitudinal study launched in 2003 to develop clinical, imaging, genetic, and biochemical biomarkers for the early detection and tracking of Alzheimer’s disease. The image query returned 13,988 records from 603 subjects, each potentially represented by multiple preprocessing variants, acquisition visits, and sequence types. Two restrictions were applied in sequence. Records were first limited to intensity-corrected native-geometry T1 volumes, which removed derived and masked variants without excluding any subject. They were then limited to the earliest routine visit codes (screening, baseline, month 6, month 12, month 24); 308 subjects had no acquisition under those codes. And this is the sole subject-level exclusion criterion. Because those codes are an ADNI-1 convention, the resulting cohort is ADNI-1 dominated. A one-row-per-subject protocol then selected a single volume for each remaining subject, eliminating leakage from repeated measurements. Selection followed a priority hierarchy favouring the most complete preprocessing available for each subject. Priority 1 combined N3 bias field correction with B1 inhomogeneity correction and gradient warping distortion correction. Where Priority 1 was unavailable, the protocol fell back to progressively less complete correction combinations (N3 + gradient warping, then B1 + gradient warping, then gradient warping alone). Baseline visits were preferred over follow-up scans and MPR sequences over MPR-R when multiple acquisitions existed for the same subject. The resulting cohort comprised 295 unique subjects, of whom 118 had an Alzheimer’s diagnosis (AD) (40%), and 177 were cognitively normal (CN) controls (60%) (Figure 1), with 83.4% receiving Priority 1 preprocessing.

The cohort was partitioned into training, validation, and test sets at the subject level using a 70/15/15 ratio. Subject-level partitioning is essential because Yagis et al. [21] demonstrated that slice-level splitting inflates classification accuracy by approximately 29% on ADNI data, as adjacent slices from the same subject share anatomy and effectively constitute data leakage. Scanner manufacturer alone is classifiable, with approximately 99% accuracy across ADNI’s 54 acquisition sites [5], so stratification was performed jointly on diagnosis and acquisition site using MultilabelStratifiedShuffleSplit from the iterstrat library with a fixed random seed of 42. The split proceeded in two stages, first separating 15% for the test set, then splitting the remaining subjects into training and validation at an 81.3/18.7 ratio. Site membership and its allocation across the three partitions are given in Table S1, and age, sex, MMSE, global CDR, APOE genotype, ADNI phase, scanner manufacturer, model and field strength are tabulated by partition and by diagnosis in Tables S2 and S3. Whether manufacturer, field strength or site can be recovered from the processed images, and whether the AD versus CN signal survives site-blocked, leave-one-manufacturer-out, single-field-strength and manufacturer-matched designs, is reported in Table S7. A chi-square test confirmed no significant class imbalance across the resulting train, validation, and test partitions (chi-squared = 3.053, *df* = 2, *p* = 0.217; Table 1b). Demographic and clinical characteristics are given by diagnostic group in Table 1a and by partition in Table 1b. Acquisition variables (site, scanner manufacturer and model, field strength, and ADNI phase) are tabulated in Appendix A; none is associated with diagnosis.

### 2.2. Preprocessing Pipeline

The preprocessing pipeline consists of six sequential stages, each producing verified outputs that serve as inputs to the next (Figure 1). A checkpoint gate follows every stage, which requires all outputs to meet predefined criteria before processing continues. This design prevents error propagation, ensuring that a quality failure at any stage is caught before it corrupts the downstream results. All 295 subjects passed every stage with a 0% rejection rate.

Brain extraction (Stage 2) removes non-brain tissue using HD-BET [23], a deep learning skull-stripping tool trained on multicentric clinical data that outperforms six established algorithms in a comparative evaluation. Running in accurate mode with test-time augmentation, HD-BET achieved a 100% success rate across all 295 volumes, with an average runtime of approximately 4 s per subject. All outputs were verified for RAS orientation and 1 mm isotropic voxel spacing.

Spatial registration (Stage 3) aligns all volumes to the FSL MNI152 T1 1 mm brain-extracted template (182 × 218 × 182 voxels) [24] using a twelve-degree-of-freedom affine transformation implemented with ANTs and SimpleITK. Registration is the most consequential preprocessing step for generative modelling. Without spatial alignment, anatomical structures occupy different voxel positions across subjects, forcing the generative model to average over misaligned features and producing blurred outputs. With registration, the diffusion model can focus its capacity on learning anatomical variation rather than spatial location. All 295 volumes were registered to a common shape of 182 × 218 × 182 voxels at 1 mm isotropic resolution, with a mean normalised cross-correlation of 0.563 (range 0.414 to 0.648). Ventricle alignment across subjects was confirmed through visual inspection. A twelve-degree-of-freedom affine removes absolute brain volume by construction, so volume cannot carry the diagnostic signal after registration. Whether ratio-based morphometry survives is quantified in Section 3.2.

Quality control (Stage 4) applies automated outlier detection across six metrics computed for every volume. These include brain volume, mean and standard deviation of intensity, mask contiguity, shape consistency, and NaN/Inf detection. Seven subjects exceeded the three-standard-deviation threshold on at least one metric. All seven were accepted after a visual review of three-plane montages confirmed the absence of genuine artefacts. The 0% rejection rate reflects the standardised acquisition protocol maintained across ADNI sites.

Anatomically guided slice extraction (Stage 5) produces twenty coronal slices per subject at twenty fixed MNI Y-coordinates (+58, +42, +26, +14, +2, −4, −8, −16, −20, −24, −32, −40, −45, −50, −55, −62, −70, −80, −90 and −100 mm), following a variable-density sampling design informed by Braak-staging neuropathology [2,25]. Seven slices are densely sampled through the hippocampal formation (Y = +2 to −32 mm at 4 to 8 mm spacing), targeting the structures that show the earliest atrophy in Braak stages III and IV, and that carry the highest discriminative value for AD classification [14]. Four additional slices capture the posterior cingulate cortex and precuneus (Y = −45 to −62 mm), regions showing early metabolic abnormalities in the Alzheimer’s continuum. The remaining nine slices provide sparse frontal and occipital reference anatomy at 10 to 16 mm intervals. Prior to slicing, each volume is normalised to [0, 1] using per-volume percentile clipping at the 0.5th and 99.5th percentiles, which is the evidence-based standard for generative brain MRI preprocessing [26]. The 99.5th percentile values ranged from 47.3 to 2271.5 across the 295 subjects, a nearly 50-fold variation in raw intensity that the normalisation successfully harmonises to a common range. Each coronal plane of the registered volume measures 182 × 182 voxels and is resampled to 256 × 256 by cubic-spline interpolation (order 3, scale factor 1.4066), with no padding or cropping, then saved as a float32 array. All twenty slice positions were verified to contain between 42% and 75% brain tissue, confirming that no slice captures predominantly empty space.

Data splitting (Stage 6) partitions the cohort into training, validation, and test sets using the subject-level stratified protocol described in Section 2.1, producing 5900 slices in total across three non-overlapping sets, with manifest files tracking each subject through the complete pipeline.

### 2.3. Model Architecture

**Figure 2:**
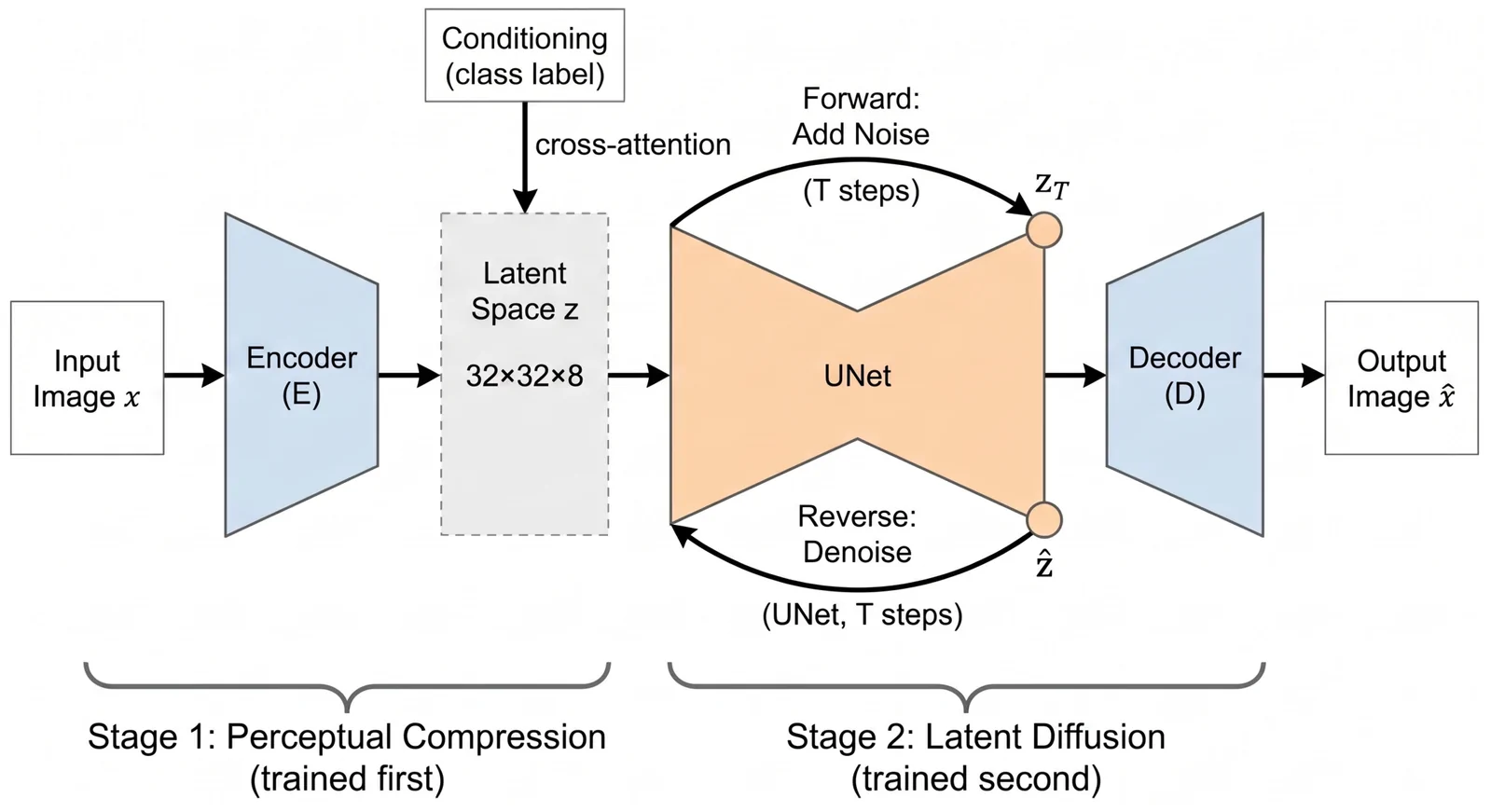
Two-stage conditional latent diffusion pipeline. In Stage 1 (perceptual compression, trained first), an encoder *E* maps an input image *x* to a compact latent representation *z* of size 32 × 32 × 8. In Stage 2 (latent diffusion, trained second), a forward process adds noise to the latent over *T* steps to reach *z*_T_, and a UNet learns the reverse process, denoising over *T* steps to recover $\hat{z}$ while conditioning on the diagnostic class label through cross-attention. A decoder *D* reconstructs the denoised latent into the output image $\hat{x}$. The dashed box marks the shared latent space linking the two stages.

The pipeline follows the two-stage latent diffusion paradigm introduced by Rombach et al. [9] and applied to medical imaging by Müller-Franzes et al. [11] as Medfusion. Stage 1 compresses input images into a compact latent representation. Stage 2 generates new latent codes conditioned on diagnostic class, which are then decoded back to pixel space (Figure 2). This separation is motivated by computational and quality considerations. Running diffusion at 256 × 256-pixel resolution would be impractical for small-dataset research on accessible hardware, whereas a 32 × 32 latent resolution reduces dimensionality by approximately 64-fold. The architecture also introduces a fundamental constraint. Autoencoder reconstruction fidelity sets an absolute ceiling on generation quality, making autoencoder design and training the first-order concern.

#### 2.3.1. Stage 1 (VAEGAN Autoencoder)

The autoencoder is implemented using MONAI’s AutoencoderKL [27] with a four-level encoder–decoder architecture. The encoder applies successive downsampling along the channel dimensions (64, 128, 256, 512), each stage containing two residual blocks, reducing the input from 256 × 256 × 1 to a 32 × 32 × 8 latent representation (downsampling factor *f* = 8). Self-attention is applied only at the deepest level, while non-local attention is enabled in both the encoder and decoder for global context aggregation. The eight-channel latent space follows the Medfusion finding that eight channels preserve fine medical structures more faithfully than the four channels used in standard natural-image autoencoders [11]. The downsampling factor of *f* = 8 was selected based on Rombach et al. [9], who demonstrated that *f* = 4 and *f* = 8 achieve the strongest trade-off between compression and perceptual fidelity.

The loss function combines five components. L1 pixel reconstruction loss (weight 1.0) provides the primary reconstruction signal. Learned perceptual loss using LPIPS with VGG-16 features (weight 0.5) penalises high-level structural differences invisible to pixel-wise metrics [17]. Multi-scale structural similarity loss (weight 0.1) preserves luminance, contrast, and structural patterns across spatial scales. KL divergence regularisation (*β* = 1 × 10^−6^) provides a deliberately minimal constraint on latent space structure, contributing only 0.000025 to the total loss at the observed raw KL of 25 and producing a near-deterministic posterior with a standard deviation of 0.0116 [28]. A PatchGAN adversarial loss with a hinge criterion (weight 0.01) sharpens reconstructions once introduced in Phase 2. The LPIPS weight of 0.5 follows the Medfusion loss configuration.

Training follows a two-phase schedule. Phase 1 (epochs 0 to 39) trains with reconstruction losses only, allowing the autoencoder to learn reasonable image reconstructions before introducing adversarial pressure. Phase 2 (epoch 40 onward) adds a three-layer PatchGAN discriminator with spectral normalisation and an R1 gradient penalty (*γ* = 10), stabilisation techniques essential for small-dataset adversarial training [7]. The optimiser was AdamW with a learning rate of 1 × 10^−4^, weight decay of 1 × 10^−4^, and gradient clipping at 1.0. Early stopping with patience of 30 epochs on validation reconstruction loss selected epoch 70 as the best checkpoint. Total training time was 3.8 h on an NVIDIA A100 GPU.

The trained latent space has a mean of −3.16, a standard deviation of 5.40, and a range of [−48.7, 34.4]. The scale factor 0.1852 (=1/5.40) normalises latents to approximately unit variance before the diffusion stage, serving as a critical constant that links the two pipeline stages.

#### 2.3.2. Stage 2 (Conditional Latent Diffusion Model)

The diffusion model uses MONAI’s DiffusionModelUNet with channel dimensions of (128, 256, 256), deliberately compact compared to Stable Diffusion’s (320, 640, 1280). This sizing reflects both the modest latent resolution of 32 × 32 and the small training set of 4080 slices. Dar et al. [29] demonstrated that smaller generative architectures memorise less patient data, making a compact design preferable for medical imaging. The network comprises three levels with self-attention at levels two and three (operating at 16 × 16 and 8 × 8 resolution), totalling approximately 39.1 million parameters. Input and output channels are set to eight, matching the autoencoder’s latent dimensionality.

Class-conditional generation is implemented via cross-attention. A learned embedding maps three tokens (CN at index 0, AD at index 1, and an unconditional token at index 2) to a 64-dimensional space. At each attention level, UNet features attend to the projected class embedding through query, key, and value projections. The unconditional token is used during training for classifier-free guidance [30]. With probability *p* = 0.15, the class label is replaced by the unconditional token, enabling the model to learn both conditional and unconditional score estimates. At inference, two forward passes per timestep produce conditioned and unconditioned predictions, combined as *ε* = *ε*_uncond + *w*(*ε*_cond − *ε*_uncond). The guidance scale *w* = 2.0 follows Dhinagar et al. [13], who used the same value for conditional brain MRI generation on ADNI. It is lower than the range of 3 to 5 that is typical for natural-image synthesis. No coordinate, anatomical label or positional encoding enters the model. The anatomical level is learned implicitly from the training distribution rather than controlled at sampling time.

The noise schedule uses a scaled linear beta schedule with *β*_start = 0.0015 and *β*_end = 0.0195 over *T* = 1000 timesteps. This wider range compared to Stable Diffusion (0.00085 to 0.012) compensates for the higher variance of the latent space (std ≈ 5.4 versus approximately 0.7 for natural image autoencoders), ensuring that the forward process reaches sufficient noise levels to enable generation from pure noise. The training objective is the simplified noise-prediction loss of Ho et al. [8], in which the model predicts the noise added at each timestep, conditioned on the noisy latent, the timestep, and the class label. Inference uses DDIM with 50 deterministic steps, matching the sampler and step count adopted by Pinaya et al. [10], who reduced the reverse process from 1000 DDPM steps to 50 DDIM steps for brain MRI generation.

Training used AdamW with a learning rate of 2.5 × 10^−5^, weight decay of 0.01, cosine decay after a 500-step warmup, and gradient clipping at 1.0. Batch size was 16 with a WeightedRandomSampler to oversample the minority AD class to approximately 50/50 per batch. Exponential moving average with a decay of 0.999 was applied to both UNet and class embedding weights for inference. Early stopping with a patience of 25 selected epoch 95 as the best checkpoint from 121 total epochs, with a validation loss of 0.0559. The training and validation loss gap stayed below 0.003 across all 121 epochs, which is consistent with limited overfitting; the memorisation analysis in Section 3.4 tests it directly. Total training time was 82.3 min on an NVIDIA A100 GPU.

The complete generation pipeline proceeds in four steps. A latent tensor is sampled from a standard normal distribution with shape 32 × 32 × 8. DDIM denoising runs for 50 steps with class conditioning to produce a denoised latent. The latent is then divided by the scale factor (0.1852) to restore the autoencoder’s native scale. Finally, the VAEGAN decoder maps the latent space back to a 256 × 256 synthetic MRI slice, with output values clipped to [0, 1]. A critical implementation detail governs this connection. The DDIM scheduler in MONAI 1.4.0 defaults to clip_sample = True, which clips denoised latents to [−1, 1] at every sampling step. Because the autoencoder’s latent space ranges from −48.7 to 34.4, this default destroys the latent distribution and produces noise rather than brain anatomy. Setting clip_sample = False resolves this completely, but it is not apparent from the training metrics alone, since the training and sampling code paths use different scheduler configurations.

### 2.4. Computational Environment

Preprocessing was performed locally on Windows 11 with WSL2 Ubuntu, on a machine with an Intel Core i9-11900H CPU (Intel Corporation, Santa Clara, CA, USA), an NVIDIA RTX 3060 notebook GPU (NVIDIA Corporation, Santa Clara, CA, USA) and 40 GB RAM, using HD-BET 2.0.1 for skull-stripping and ANTsPy 0.6.2, SimpleITK 2.5.3 and NiBabel 5.3.3 for registration, resampling and file handling. Model training and evaluation were performed on Google Colab Pro. The VAEGAN was trained on an NVIDIA A100-SXM4-40 GB GPU (NVIDIA Corporation, Santa Clara, CA, USA; 3.8 h). All subsequent trainings and evaluations were run on an NVIDIA L4 GPU, including the diffusion model (82.3 min, 4.7 GB peak VRAM). The software environment was Python 3.12, PyTorch 2.9.0 with CUDA 12.8, MONAI 1.4.0, NumPy 1.26.4 and scikit-learn 1.8.0; exact package versions are recorded in the repository. The data split, diffusion training, sampling and downstream classifier used a fixed seed of 42. The VAEGAN was trained without a fixed seed, so stage 1 is not bit-reproducible from source, and the released checkpoint is authoritative for it.

### 2.5. Evaluation Framework

Evaluation follows a four-tier protocol designed for small-cohort synthesis, where standard metrics have known limitations.

Tier 1 assesses autoencoder reconstruction quality using SSIM, PSNR, and LPIPS [17], establishing the quality ceiling for generation. Because any loss of detail at this stage propagates irreversibly to generated images, reconstruction metrics must exceed their targets before proceeding to diffusion model evaluation.

Tier 2 measures distributional similarity between the real and generated image sets. KID [20] is the primary metric because it is an unbiased polynomial MMD^2^ estimator that avoids the O(1/*N*) upward bias that affects FID at small sample sizes [19]. It is computed over 100 random subsets of 200 images each under a fixed seed and reported as the mean and standard deviation across subsets. FID is reported secondarily using the FID_∞_ extrapolation method, in which FID is computed at six sample sizes (*N* = 200, 350, 500, 650, 800, 880), with ten independent draws at each size except the largest, where the pool is exhausted and only a single draw exists, and extrapolated to N approaching infinity by ordinary least-squares regression of FID on 1/*N*, whose intercept is the bias-corrected estimate. Its 95% interval is obtained from 2000 bootstrap resamples of the generated set. Precision and recall [31] provide complementary information, separately quantifying fidelity (the fraction of generated images falling within the real-data manifold) and diversity (the fraction of the real distribution covered by generated images). Both use *k* = 3 nearest neighbours, with the manifold taken as the union of all reference hyperspheres. All distributional metrics are computed in Inception v3 feature space, which is trained on natural images; the values reported below are specific to that extractor and are not absolute measures of image quality. DINOv2 features were also evaluated and produced FID values on an entirely different scale. Distances computed in different embeddings are not commensurable in either direction, and no ranking of the two extractors is implied. DINOv2 is retained only for memorisation detection (Tier 3), where the Scardace et al. [18] framework was originally validated. Whether an image is flagged depends only on whether it lies nearer to the training set than to the held-out set, a comparison of the ordering of two distances rather than their absolute scale, and is, therefore, far more robust to the feature-space distortion that inflates DINOv2-based FID than a distributional distance would be. Because the real-image control is computed in the same space, any systematic component of that distortion shifts the generated and control distributions together and is absorbed in the gap between them.

Tier 3 assesses memorisation using the Scardace et al. [18] nearest-neighbour ratio metric, with two critical additions that are absent from the original methodology. Training images are subsampled to *N* = 880 to match the holdout set size, eliminating a set-size confound that inflates apparent memorisation when the reference sets are unequal. The identical metric is also run on real test images as a leave-one-out control, establishing a null baseline flagging rate. Severity is graded into three categories based on the nearest-neighbour ratio. Values below 0.5 indicate instance memorisation (near-copies of the training data), values between 0.5 and 1.0 indicate distributional proximity (closer to the training data than to the holdout set, which is expected for well-trained models), and values at or above 1.0 indicate distributional generalisation.

Tier 4 evaluates downstream classification performance through the Train-on-Synthetic-Test-on-Real (TSTR) paradigm. A ResNet-18 classifier [32] is trained exclusively on synthetic images and evaluated on real test data, with a corresponding Train-on-Real-Test-on-Real (TRTR) experiment providing the upper bound. The classifier performs no synthesis; it is a downstream probe of how much diagnostic signal the generated images carry. The synthetic training set comprises 4080 slices, 1520 AD and 2560 CN, matching the class proportions of the real training split rather than balancing them. Both arms use ImageNet-pretrained weights with a new single-logit head and no frozen layers, binary cross-entropy loss, AdamW at a learning rate of 1 × 10^−4^ with weight decay 0.01, cosine annealing, batch size 32, at most 30 epochs, and no augmentation. Checkpoints are selected by early stopping on the real validation split (940 slices, 47 subjects, patience 10), which the synthetic arm, therefore, sees for model selection but never for a gradient update. The real test split (880 slices from 44 subjects, 18 AD and 26 CN) is used for no selection of any kind. To separate sampling variance from initialisation variance, each arm is run ten times per architecture across three independent synthetic samplings, and the whole protocol is repeated with DenseNet-121 to test whether the conclusion depends on the evaluator. Subject-level aggregation is performed by averaging the predicted probabilities across the twenty slices per subject; a correction is required because Yagis et al. [21] demonstrated that slice-level evaluation inflates AUC by approximately 29% on ADNI data. Because the twenty slices from one participant are not independent, uncertainty is quantified at the subject level. Ninety-five percent confidence intervals come from a stratified cluster bootstrap [33] over 5000 replicates, in which the 18 AD and 26 CN test subjects are resampled separately with replacement and each subject’s slices are taken as a whole cluster. Paired comparisons between arms use DeLong’s test on the subject-level scores together with a paired subject bootstrap [34,35].

### 2.6. Baseline Comparison and Preprocessing Ablation

#### 2.6.1. Uncontrolled Pipeline Comparison

Two pipeline configurations were developed on the same 295 ADNI participants. The baseline pipeline (v1) worked in native skull-stripped subject space without spatial registration and selected twenty slices per participant by linear spacing across the central 35 to 65 per cent of that participant’s own brain extent, so that a given slice index corresponded to a different anatomical level in different participants. The enhanced pipeline (v2) registered every volume to MNI152 space and sampled twenty fixed coordinates shared by all participants. The two configurations also differ in intensity normalisation, in the method used to resample volumes to 256 × 256, in latent dimensionality, in perceptual and adversarial loss configuration, in the diffusion architecture and its conditioning mechanism, in the sampler settings applied at evaluation, and in the subject split. Any difference in output between them, therefore, reflects a complete pipeline redesign and cannot be attributed to preprocessing. The two configurations were never evaluated under a common harness, so no numerical comparison between them is reported. The full list of differences between the two configurations is given in Appendix A, Table A1.

#### 2.6.2. Controlled Preprocessing Ablation

To isolate the contribution of preprocessing, a controlled ablation was run in which the enhanced architecture, losses, training schedule, sampler and evaluation harness were held fixed and only the preprocessing of the input data was varied. One arm was trained on slices produced by the baseline preprocessing and the other on slices produced by the enhanced preprocessing. Both used the enhanced subject split, re-applied by participant identifier, so that no participant crossed between partitions in either arm. The latent scale factor is a derived quantity rather than a hyperparameter and was recomputed for each arm, giving 0.2367 and 0.1852, respectively. Generation used common random numbers across the two arms and produced 880 images per arm, 360 AD and 520 CN, at guidance scale 2.0 with 50 DDIM sampling steps.

Both arms were scored with the metric harness described in Section 2.5. Uncertainty on the between-arm difference was estimated by a paired subject-level bootstrap over the 44 test participants, using 1000 replicates for FID, 400 for KID and 500 for precision and recall. Precision and recall use m-out-of-n resampling without replacement with *m* = 30 [33], and their intervals are reported without the corresponding variance rescaling, which makes them conservative rather than optimistic.

Three residual limitations of this ablation are stated here rather than in the Results. Seventy of the 295 participants contributed a different ADNI image to the archived baseline slices, 55 of them from a different visit, so a small image-level difference persists alongside the preprocessing manipulation. The enhanced arm reuses checkpoints trained before the seeding regime applied to the baseline arm was adopted. Each arm was trained once, so the reported intervals cover sampling variance and not training-run variance.

## 3. Results

### 3.1. Reconstruction Quality

**Figure 3:**
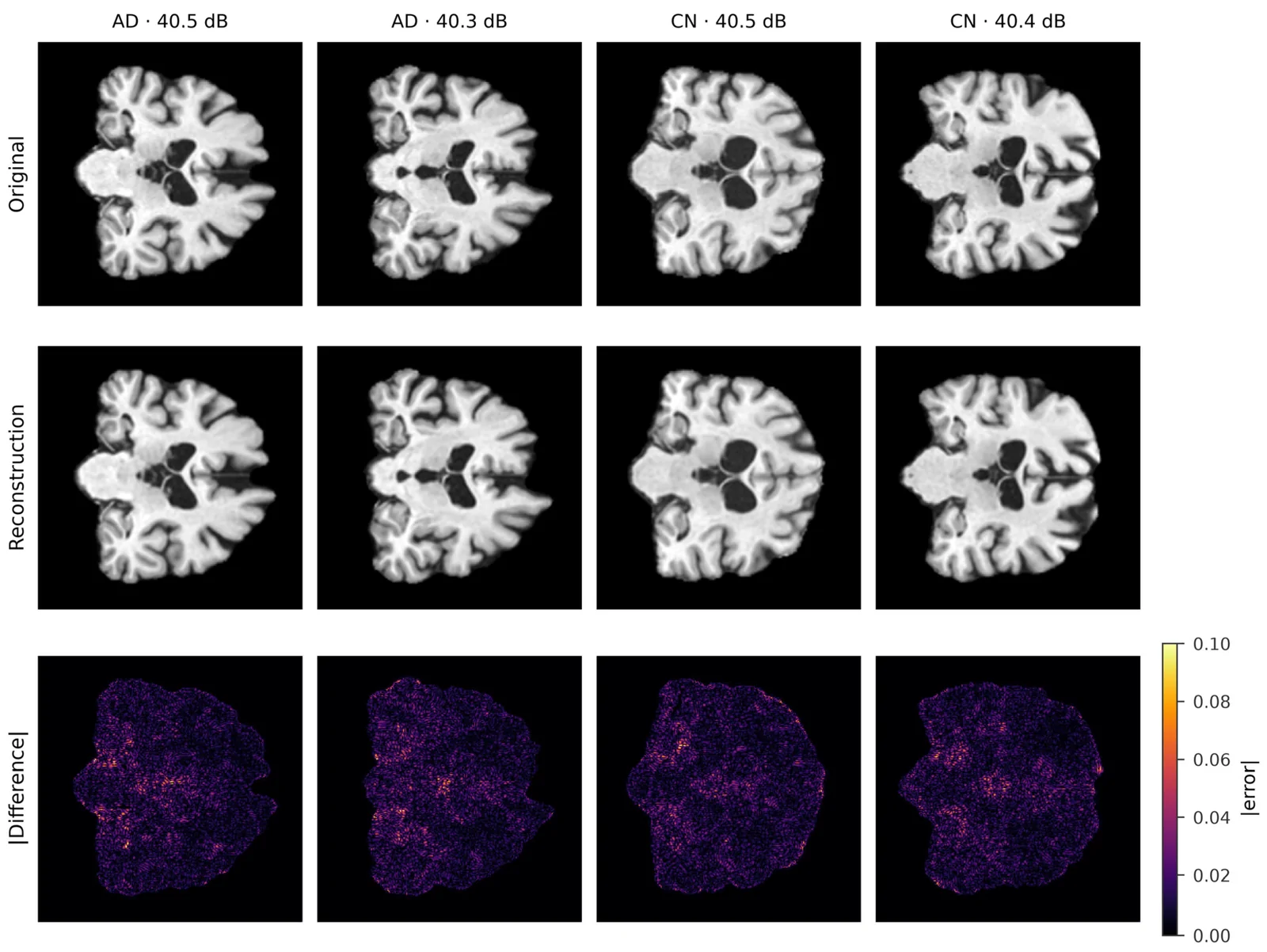
Autoencoder reconstruction quality on held-out test slices. Each column shows one example mid-brain slice at the hippocampal level, giving two Alzheimer’s disease (AD) cases and two cognitively normal (CN) cases. Rows show the original (**top**), reconstruction (**middle**), and absolute per-pixel difference (**bottom**), with difference maps windowed to [0, 0.10] in normalised-intensity units. Each column header reports the peak signal-to-noise ratio (PSNR) of that slice. Over the full test set (*n* = 880 slices), the autoencoder reached a mean PSNR of 38.66 dB and a mean structural similarity (SSIM) of 0.989, and reconstruction error stayed low for both classes with no visible class-dependent bias.

The VAEGAN autoencoder was selected at epoch 70, the epoch with the lowest validation reconstruction loss. On the validation split, it reached a PSNR of 38.44 dB against a target of 32 dB, an SSIM of 0.9861 against a target of 0.95, and an LPIPS of 0.0166 against a target of 0.08. All values in this paragraph are validation-split values; the corresponding test-split values are reported in Figure 3. Visual inspection of the reconstructions at epoch 70 confirmed that anatomical structures, including sulci, ventricles, grey–white matter boundaries, and the cortical ribbon, were faithfully preserved. AD-specific features such as ventricular enlargement and temporal horn prominence were accurately reproduced, as were CN-specific features including compact ventricles and preserved cortical thickness (Figure 3).

Training dynamics validated the two-phase schedule. Phase 1 reached a PSNR of approximately 35.6 dB before the PatchGAN discriminator was introduced at epoch 40. The discriminator loss spiked to 253.9 at Phase 2 onset, as expected when a discriminator encounters reconstructions already close to real images, then converged to equilibrium at 1.0 by epoch 50. Validation PSNR fluctuated after epoch 70 without a sustained trend, ranging from 34.29 dB at epoch 90 to 38.20 dB at epoch 95, so the training curve does not by itself demonstrate degradation. Epoch 70 was selected because it gave the lowest validation reconstruction loss. Reconstruction quality by coronal level is given in Table S5. These reconstruction metrics set the quality ceiling for the subsequent generation stage.

### 3.2. Qualitative Appearance

**Figure 4:**
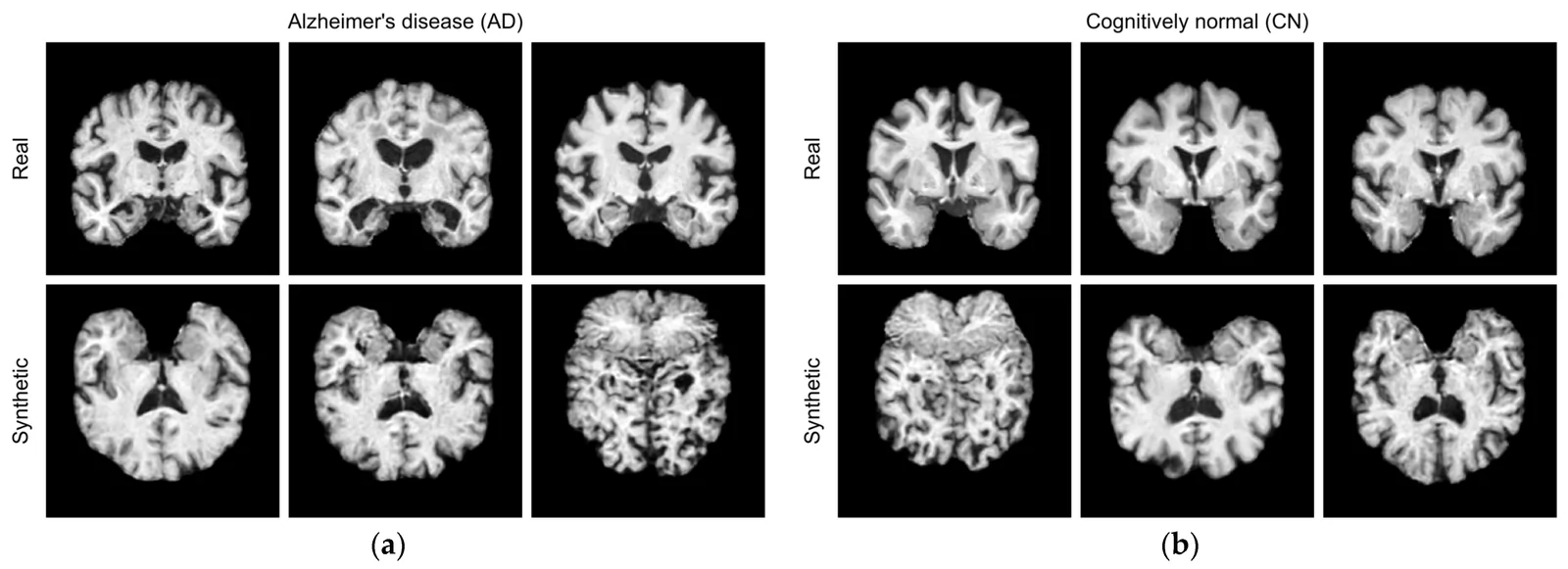
Real and synthetic coronal brain MRI slices by diagnostic class. (**a**) Alzheimer’s disease (AD); (**b**) cognitively normal (CN). In each panel, the top row shows real held-out test slices, and the bottom row shows synthetic slices from the enhanced pipeline, selected as median-typical generations by brain-area fraction rather than for visual quality. Columns are not paired; each synthetic image is sampled independently and corresponds neither to the real slice above it nor to any individual participant. Because the model is conditioned on diagnosis alone and not on anatomical level (Section 2.3.2), synthetic slices are not matched to the coronal level of the real slices above them. Synthetic images reproduce class-characteristic gross morphology, with larger ventricles and wider sulci in AD than in CN, while fine cortical detail is resolved less sharply than in the real slices.

The synthetic images capture the class-defining gross morphology (Figure 4): enlarged lateral ventricles and widened cortical sulci in the Alzheimer’s disease cases and compact ventricles with a fuller cortical mantle in the cognitively normal cases. Fine cortical detail is resolved less sharply than in the real slices, and occasional generations show an incomplete or asymmetric structure. The class contrast is not left to visual impression. In a probe sample of 32 generations per class, a ventricular-area proxy was higher for AD than for CN (0.173 against 0.124, Cohen’s *d* = 0.88). These are the authors’ own observations. No blinded expert reader study was conducted, and no claim in this paper rests on them. The separation between well-formed class-level anatomy and imperfect fine detail frames the quantitative results that follow. The distributional metrics in Section 3.3 measure the residual gap, and the precision of 0.272 reported in Table 2 quantifies the fraction of samples lying within the real-image manifold.

**Table 2:** Distributional generation-quality metrics for the enhanced pipeline, computed on 880 generated images against the held-out test set using Inception v3 features. KID and FID are lower-is-better; precision and recall are higher-is-better. KID is reported as the mean ± standard deviation over 100 random subsets. FID_∞_ is the bias-corrected value obtained by extrapolating FID to infinite sample size [19] (Figure 5).

Metric	Value
Kernel Inception Distance (KID), overall	0.030 ± 0.002
Fréchet Inception Distance (FID), raw (*N* = 880)	48.49
FID_∞_ (bias-corrected)	43.82 (95% CI 43.24–44.40)
Precision	0.272
Recall	0.417

**Figure 5:**
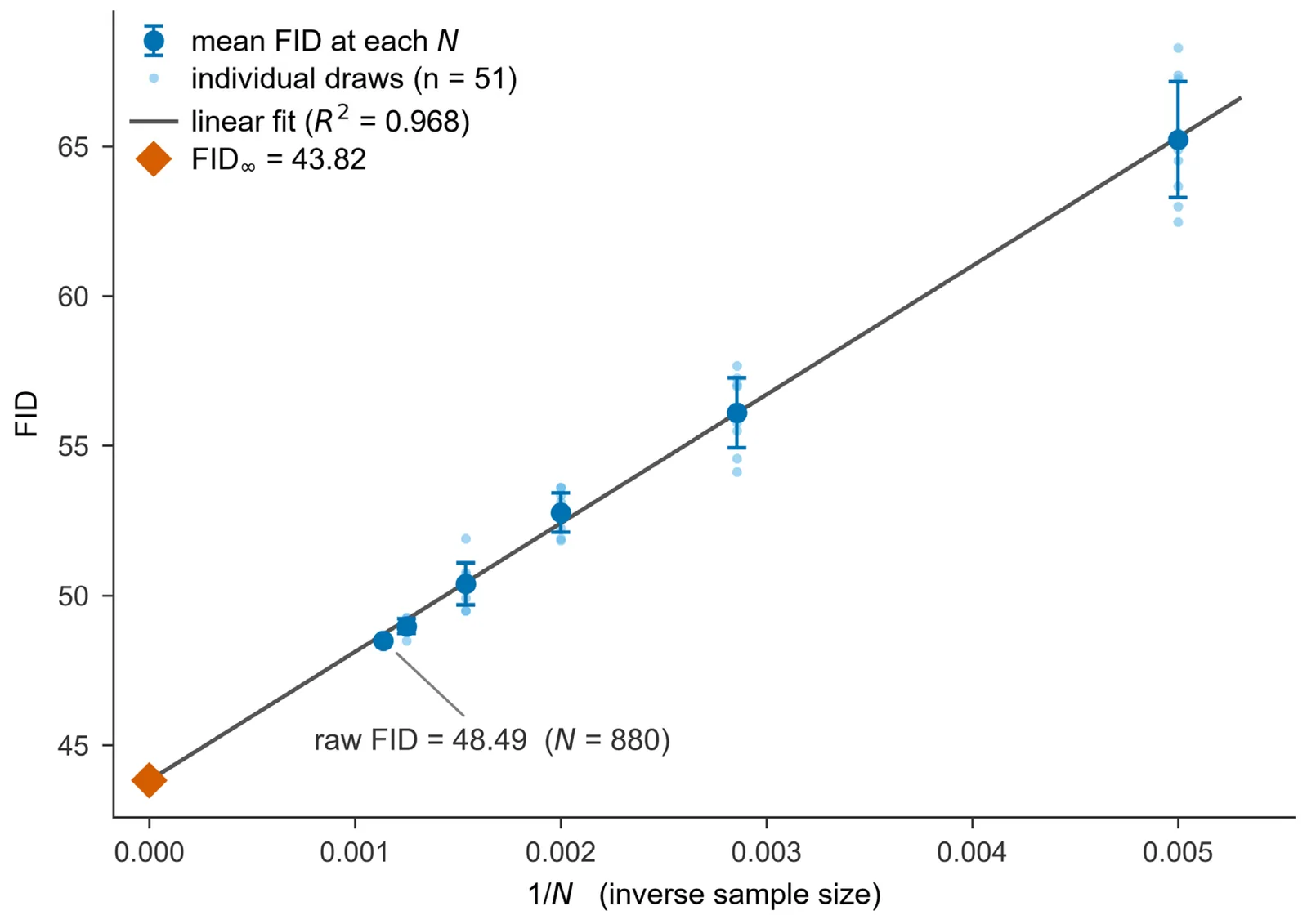
Bias-corrected FID via sample-size extrapolation. Fréchet Inception Distance (FID) was computed at six sample sizes (*N* = 200, 350, 500, 650, 800, 880) and regressed against 1/*N* following the extrapolation method of Chong and Forsyth [19]; the intercept at 1/*N* → 0 gives the bias-free estimate FID_∞_. Dark circles show the mean FID at each N over ten independent draws, with error bars giving ±1 standard deviation across those draws; the largest sample exhausts the generated pool and so admits a single draw. Light points are the individual draws (51 in total). The grey line is the linear fit (*R*^2^ = 0.968), and the orange diamond marks FID_∞_ = 43.82. The raw FID at the largest sample (*N* = 880) was 48.49, so the extrapolation removes about 4.7 points of upward small-sample bias.

### 3.3. Distributional Metrics

The primary distributional metric, Inception v3 KID, measured 0.030 ± 0.002 overall for the enhanced pipeline (Table 2). This value is specific to the Inception v3 feature space and carries no absolute interpretation; we are not aware of a validated reference range for KID in medical image synthesis, and none is assumed here.

The raw FID at *N* = 880 was 48.49, but this value overestimates the true distributional distance due to FID’s O(1/*N*) sample-size bias [19]. FID_∞_ extrapolation, computed at six sample sizes and regressed against 1/*N*, yielded a bias-corrected estimate of 43.82 with *R*^2^ = 0.968 and a bootstrap 95% interval of 43.24 to 44.40 (Figure 5). The fit is consistent with the 1/*N* bias model and indicates that the raw value overestimates FID_∞_ by approximately 4.7 points, a relative bias of about 10%. This interval describes the uncertainty of the extrapolation itself and not subject-level sampling variability. No comparison is drawn with the baseline configuration, for the reasons given in Section 2.6.1. Per-class raw FIDs of 56.03 for AD and 60.44 for CN were both higher than the overall value, consistent with bias amplification when 880 samples are split into smaller class-specific subsets.

Inception precision was 0.272 and recall was 0.417. The pattern of precision falling below recall is typical of models operating through a spatial bottleneck. The 32 × 32 latent space captures the general anatomical structure effectively, producing good coverage of the real distribution (recall 0.417, meaning 42% of the real distribution is represented) but smooths fine details that would place the generated images precisely within the real-data manifold (precision 0.272, meaning 27% of generated images fall within the real-image manifold). One possible interpretation is that the primary quality limitation is autoencoder compression rather than diffusion model capacity, although the present data do not distinguish between the two.

### 3.4. Memorisation Analysis

None of the 880 generated images exhibited instance memorisation. No image produced a nearest-neighbour ratio below 0.5 in DINOv2 feature space, the threshold below which a generated image would resemble a near-copy of a specific training example. True instance memorisation typically produces ratios of 0.05 to 0.30. The worst-case generated image had a ratio of 0.766, far above this range.

**Figure 6:**
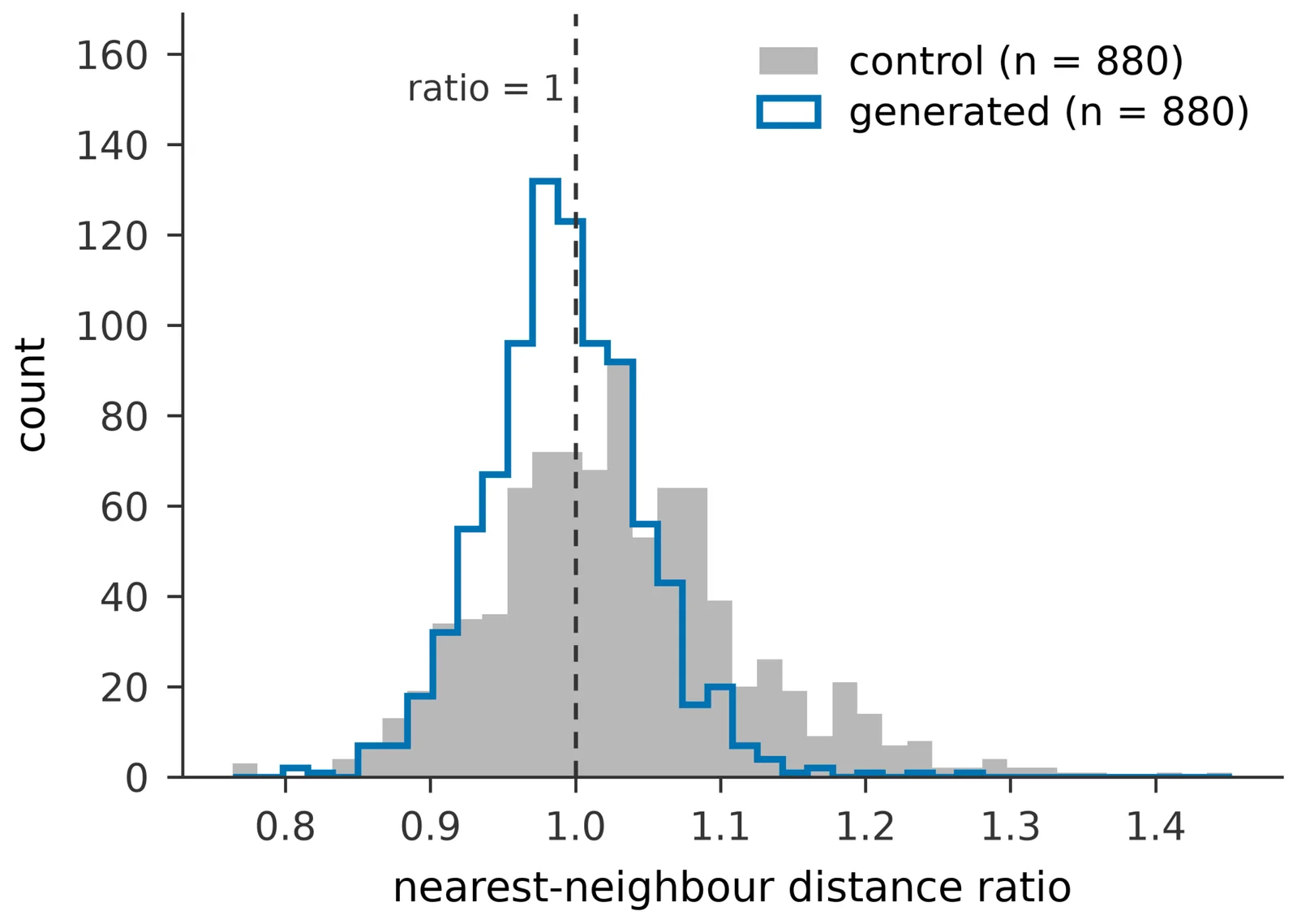
Nearest-neighbour distance-ratio distributions for the memorisation control analysis. For each image, the ratio is the distance to its nearest training neighbour divided by the distance to its nearest held-out test neighbour in the DINOv2 feature space [18]; a ratio below 1 (dashed line) indicates an image lying closer to the training set than to held-out data. The grey distribution is a size-matched real-image control (*n* = 880) that establishes the expected baseline, and the blue outline is the generated set (*n* = 880). The generated distribution is shifted towards lower ratios: 63.5% fall below 1, compared with 38.5% for the control, a gap of 25.0 percentage points. No sample fell below 0.5, and no instance-level match was found, indicating distributional proximity to the training data without verbatim copying.

The uncorrected flagging rate, computed with the original unequal reference sets (4080 training images versus 880 test images), was 91.25%. A control experiment applying the same metric to real test images under equal-sized reference conditions (880 by 880) revealed a baseline flagging rate of 38.5%. This control establishes that the Scardace et al. [18] metric produces substantial artefactual flagging from set-size imbalance alone. After correcting by equalising the reference set sizes, the generated flagging rate dropped to 63.5%, a gap of 25.0 percentage points above the control baseline (Figure 6). The difference between that naive rate and the corrected rate is 27.7 percentage points, and the correction consists solely of shrinking the training reference from 4080 images to 880. So, all of it is attributable to set size. The corrected estimate is robust to the subsample. Across 1000 independent training subsamples, the flagging rate stayed within a 95% interval of 57 to 72 percentage points, the generated-minus-control gap remained positive in every subsample (95% interval 16 to 32 percentage points), and no generated image was flagged as instance memorisation in any subsample.

The distribution of generated ratios was unimodal and centred at a mean of 0.985, with a range of [0.766, 1.228]. The absence of a bimodal cluster rules out the presence of a subpopulation of memorised copies hidden within the overall distribution. The eight generated images closest to the training set are shown against their nearest training neighbours in Figure S2. At the minimum ratio of 0.766, the pairs remain visibly different images. Across all 880 generated images, with nearest neighbours identified in the DINOv2 feature space, the mean per-image LPIPS distance to the nearest training image was 0.276 ± 0.100 and the mean SSIM 0.613 ± 0.136. The corresponding distances to the nearest held-out test image were 0.279 ± 0.098 and 0.613 ± 0.134; the margin between the two references is under a tenth of the between-image standard deviation in either measure. The nearest neighbours also agree on anatomy. For 54% of the generated images, the nearest training and the nearest test neighbour lie at the same coronal level, and for 80% within one level, against 6% and 14% under random pairing, so the anatomical level of a generated image is identified consistently by two independent sets of real images. For comparison, autoencoder reconstructions achieve an SSIM of 0.986 and an LPIPS of 0.017 against their own input images, placing the generated-to-nearest-neighbour distances firmly in the “different images sharing anatomical characteristics” range rather than the “near-copies” range. The severity classification places all 880 samples outside the instance-memorisation band (Table 3). This conclusion is conditional on one feature extractor, one flagging threshold and a subsampled training reference; Figure S1 shows how the flagging rate varies with the threshold and does not establish the absence of memorisation under a different embedding or a different threshold.

### 3.5. Downstream Classification Performance

Across ten training runs spanning three independent synthetic samplings, a ResNet-18 classifier trained exclusively on synthetic data reached a subject-level AUC of 0.779 ± 0.031 on the 44 real test participants; each participant scored as the mean predicted probability across its twenty slices. The Train-on-Real-Test-on-Real upper bound over the same ten runs was 0.869 ± 0.027. The difference of 9.0 percentage points is not distinguishable from zero at this test-set size. The stratified subject cluster bootstrap gives a 95% interval of −2.8 to 25.4 percentage points (*p* = 0.16), and a paired DeLong test on the seed-averaged subject scores gives *p* = 0.09. Repeating the whole protocol with DenseNet-121 gave the same picture, with TSTR 0.790 ± 0.019, TRTR 0.875 ± 0.022 and a difference of 8.5 percentage points (95% interval −2.4 to 20.5, *p* = 0.11), so the conclusion does not depend on the choice of evaluator. Decomposing the TSTR variance shows that most of it arises from classifier initialisation rather than from which synthetic sample was drawn (within-draw standard deviation 0.034 against between-draw 0.007).

Slice-level AUCs are not reported, since the twenty slices from one participant are not independent and subject-level evaluation is the valid unit following Yagis et al. [21] correction for slice-level inflation. Per-class distributional metrics were not computed under the present evaluation harness, so no class-specific claim about generation fidelity is made here.

### 3.6. Preprocessing Ablation

Preprocessing improved two of the four distributional measures. KID fell from 0.043 ± 0.004 under the baseline preprocessing to 0.028 ± 0.002 under the enhanced preprocessing, a paired difference of −0.0147 with a 95% interval of −0.0231 to −0.0080. Precision rose from 0.213 to 0.258; the paired subject-level bootstrap places the median difference at +0.069, with a 95% interval of +0.004 to +0.123. Both intervals exclude zero (Table 4).

The remaining measures did not separate the two arms. FID fell from 50.33 to 46.92, but its paired interval spans zero (−8.73 to +2.65). And recall was effectively unchanged at 0.380 against 0.385 (−0.027 to +0.043). A downstream classifier trained on each arm’s output and evaluated on real data gave slice-level AUCs of 0.669 and 0.609, a difference of −0.062 with an interval of −0.140 to +0.011. This probe uses logistic regression on Inception features rather than the ResNet-18 evaluator of Section 3.5 and is not comparable with the values reported there. No measure significantly favoured the baseline preprocessing; the downstream probe is the only measure whose point estimate leaned that way, and its interval spans zero.

KID is the primary distributional metric in this study (Section 2.5), and it separated the two arms, as did precision. FID, reported secondarily, did not. Because the ablation holds the architecture, losses, training schedule, subject split, sampler and evaluation harness fixed, the differences it detects are attributable to preprocessing within the limits stated in Section 2.6.2. The enhanced-arm values here differ slightly from those in Table 2 because the ablation generated and scored its own image set rather than reusing the evaluation set of Section 3.3. Each arm was trained once, so the intervals cover sampling variance and not training-run variance. And the ablation does not decompose the preprocessing block into registration, slice selection, intensity normalisation and resampling.

## 4. Discussion

The comparison between the baseline and enhanced pipelines cannot isolate the effect of preprocessing. The two configurations differ in registration, slice selection, intensity normalisation, resampling, latent dimensionality, perceptual and adversarial loss configuration, diffusion architecture and conditioning mechanism, sampler settings and subject split, and they were never evaluated under a common harness (Section 2.6.1). The controlled experiment in this study is instead the preprocessing ablation of Section 3.6, which held the enhanced architecture, losses, training schedule, subject split, sampler and evaluation harness fixed and varied only the preprocessing of the input data. That ablation improved KID by 0.0147 and precision by 0.069, both with intervals excluding zero, while FID, recall, and the downstream probe did not separate the arms. Registration remains the most plausible single contributor on mechanistic grounds because, without spatial alignment, anatomical structures occupy different voxel positions across subjects, forcing the diffusion model to average over misaligned features and blurring its outputs. Separating its contribution from the other preprocessing steps would require a factorial design.

The TSTR result requires careful interpretation. A subject-level AUC of 0.779 ± 0.031 from 295 subjects is a strong outcome, though estimated from only 44 test participants, and the TSTR measures downstream classification performance rather than anatomical realism. A model could theoretically score well on the TSTR by preserving the gross ventricular differences between AD and CN while generating anatomically implausible cortical detail because the classifier focuses on the most discriminative features rather than overall image quality. Integrated-gradient attribution over the real test set argues against that reading. Both classifiers place more than 99% of the attribution mass inside the brain mask, deplete the 12-pixel border band roughly 140-fold relative to its area, and distribute attribution almost identically across ventricular and hippocampal regions (Table S8). The combination of TSTR with KID, precision and recall, visual inspection, and memorisation analysis provides substantially stronger evidence than any individual metric, consistent with the multi-metric evaluation framework recommended by Deo et al. [36]. The autoencoder quality ceiling (SSIM 0.9861) is not fully exploited by the diffusion model, as the precision of 0.272 suggests that fine anatomical details are partially smoothed during generation. This gap indicates room for improvement in the generative stage, likely through additional training data rather than architectural changes.

Direct comparison with published brain MRI synthesis studies is complicated by differences in cohort size, evaluation methodology, feature extractors, and preprocessing. Müller-Franzes et al. [11] reported Medfusion FID values between 11.63 and 30.03 across three non-brain modalities with datasets of 19,958 to 223,414 images. The FID_∞_ of 43.82 achieved here on 880 test images from 295 subjects is higher than expected, given the substantially smaller training set, but still falls within a reasonable range for the data scale. Medfusion’s precision ranged from 0.66 to 0.70 across those datasets, compared to 0.272 in this project, reflecting the more constrained generative capacity available from 4080 training slices and the 32 × 32 spatial bottleneck. Dhinagar et al. [13] demonstrated a 3-percentage-point AUC improvement when augmenting AD classification with LDM-generated synthetic data from 4098 ADNI scans, approximately 14 times the cohort available here. The standalone TSTR AUC of 0.779 ± 0.031 was obtained without any real-data augmentation. Dar et al. [29] reported an average memorisation rate of 37.2% across medical imaging datasets using a contrastive copy detection framework. This project identified no near-copy instances under a nearest-neighbour ratio metric with set-size controls and the stated threshold, though the two studies employ different detection methodologies and definitions of memorisation, making direct numerical comparison inappropriate. The comparison spans cohorts from 295 to 31,740 subjects under differing architectures and protocols, and only this study and Dhinagar et al. [13] condition generation on diagnosis (Table 5).

The memorisation analysis produced a finding with implications beyond this specific pipeline. The Scardace et al. [18] nearest-neighbour ratio metric, applied without correction using unequal reference sets (4080 versus 880), flagged 91.25% of generated images. The control experiment on real test images under equal-sized reference conditions revealed a baseline flagging rate of 38.5%. After correction, which removes 27.7 percentage points of artefactual flagging, the generated rate dropped to 63.5%, a gap of 25.0 percentage points above the control. Zero images met the threshold for instance memorisation (ratio below 0.5), and the minimum observed ratio of 0.766 falls far above the 0.05 to 0.30 range characteristic of genuine pixel-level copying. The metric cannot distinguish distributional proximity from instance memorisation without severity analysis, and the recommended three-part reporting protocol (equal-sized reference sets, real-image control baseline, and severity grading rather than headline flagging percentage) is straightforward to implement and should be standard practice for small-cohort evaluation.

Ten limitations constrain the interpretation of these results. The two-dimensional slice-based generation approach produces twenty independent coronal slices per subject without enforcing volumetric consistency, meaning a generated subject may exhibit inconsistent anatomy across adjacent slices and limiting clinical application to per-slice analysis. The cohort of 295 subjects operates at the lower bound for diffusion model training, and Bonnaire et al. [37] showed that the memorisation onset timescale scales linearly with the dataset size, placing this cohort in an elevated-risk regime. The empirical evidence (zero severe cases) suggests the risk did not materialise with the chosen architecture, but this cannot be assumed for larger or differently structured models. The conditioning is limited to a binary AD versus CN distinction, whereas real diagnostic utility would require finer-grained conditioning on clinical variables such as CDR score, hippocampal volume, or disease duration. No anatomical-level information is conditioned either. Neither the coronal level nor the orientation of a slice is specified or constrained at sampling time. The ADNI cohort over-represents white, educated North American participants, and a small number of participants were scanned in ADNI-3 rather than ADNI-1. A sensitivity analysis excluding them is given in Table S4, and the results may not generalise to other populations or acquisition protocols. The cohort was not matched on demographic variables, and a class-conditional generator learns the joint distribution of the label and everything correlated with it in this cohort. So, a difference between AD-conditioned and CN-conditioned samples is not an isolated estimate of disease-related anatomy. Acquisition confounding is likewise not excluded. Participants were scanned at 54 sites on several scanner models and field strengths, and splitting jointly on diagnosis and site controls the composition of the partitions but not scanner effects on the images themselves. A site-held-out evaluation would be the appropriate test and requires a larger cohort than this one, in which the median site contributes about five participants and several contribute only one class. Inception v3 features, used for KID and FID computation, were trained on natural images and may not capture all diagnostically relevant characteristics of brain MRI. No domain-specific extractor such as RadImageNet [38] was evaluated, and every distributional value reported here is, therefore, specific to one feature space. No blinded expert reader study was conducted. All visual assessments were made by the authors rather than by a clinical expert, so no claim of anatomical realism in this paper rests on expert judgement. The generator does not fix superior–inferior orientation. A template test against the mean training image at each slice level classifies 437 of the 880 generated images (49.7%) as inverted relative to the stored orientation and all 880 real test slices as upright, consistent with the training-time flip acting along the stored-array axis corresponding to the superior–inferior direction. The memorisation result is unaffected, with mean nearest-neighbour ratios of 0.984 for upright and 0.987 for inverted images and no severe cases in either. The v1-to-v2 comparison varied many factors at once, and the two configurations were never evaluated under a common harness. So, it cannot attribute any difference in output to a component. The controlled preprocessing ablation of Section 3.6 addresses this for preprocessing as a block but does not decompose that block into registration, slice selection, intensity normalisation and resampling; a factorial design varying each step individually would be needed to attribute the improvement to any single one.

Future work should pursue five directions. Extending to three-dimensional volumetric generation using 3D latent diffusion models [10,12] would enforce inter-slice consistency, enabling volumetric clinical assessment. Replacing the DDPM formulation with flow matching [39] would reduce inference time by five- to tenfold while maintaining equivalent generation quality, representing the highest-value architectural improvement for practical deployment. Finer-grained conditioning on continuous clinical variables would produce a severity spectrum rather than a binary distinction, and the existing cross-attention mechanism can accommodate this with minimal architectural modifications. Applying the preprocessing pipeline to non-ADNI datasets such as OASIS and AIBL would test the generalisability of both the methodology and the preprocessing-matters finding across different acquisition protocols and demographics. Adopting FID_∞_ extrapolation as standard practice and conducting formal radiologist reader studies would strengthen evaluation methodology for small-cohort synthesis more broadly.

## 5. Conclusions

This study developed and evaluated a two-stage conditional latent diffusion model pipeline for generating synthetic 2D coronal brain MRI conditioned on Alzheimer’s disease diagnosis, trained on data from 295 subjects of the ADNI database. The central experiment is a controlled preprocessing ablation in which the architecture, losses, training schedule, subject split, sampler and evaluation harness were held fixed, and only the preprocessing of the input data varied.

The enhanced pipeline, incorporating MNI152 registration, variable-density anatomical slice selection informed by Braak-staging neuropathology, and literature-informed loss configuration, achieved a KID of 0.030 ± 0.002 and an FID_∞_ of 43.82, producing synthetic images that capture class-characteristic gross morphology from a cohort approximately one fourteenth the size used by the nearest comparable study. A classifier trained exclusively on synthetic data reached a subject-level AUC of 0.779 ± 0.031 against 0.869 ± 0.027 for real data, a difference not distinguishable from zero at this test-set size. The controlled preprocessing ablation improved KID and precision, both with intervals excluding zero, and no measure favoured the baseline preprocessing.

The memorisation analysis identified no near-copy instances among 880 generated samples under the feature extractor and threshold applied and quantified a 27.7-percentage-point set-size confound in the nearest-neighbour ratio metric. The corrected three-part reporting protocol (equal-sized reference sets, real-image control baseline, and severity grading) provides a practical contribution to evaluation methodology that extends beyond this specific pipeline.

These results demonstrate that rigorous, anatomically informed preprocessing can partially compensate for small cohort size in brain MRI synthesis and that preprocessing methodology deserves the same documentation and experimental attention as model architecture. Extending this work to three-dimensional volumetric generation, replacing the DDPM formulation with flow matching for faster inference, and validating the preprocessing-matters finding across non-ADNI cohorts represent the most consequential next steps towards clinical deployment of synthetic brain MRI for Alzheimer’s disease research.

## Figures and Tables

**Table 1:** Cohort characteristics. (**a**) By diagnostic group. (**b**) By data partition.

(**a**)					
**Variable**	**All (*n* = 295)**	**AD (*n* = 118)**	**CN (*n* = 177)**	***p***	**Effect Size**
Age at selected scan (y)	73.54 ± 4.25	72.73 ± 4.89	74.08 ± 3.68	0.012	*d* = −0.320
Sex, F/M	147/148	58/60	89/88	0.849	*V* = 0.011
MMSE total	26.64 ± 3.55	22.95 ± 2.54	29.19 ± 0.96	<0.001	*d* = −3.504
Global CDR	0.31 ± 0.42	0.76 ± 0.30	0.00 ± 0.04	<0.001	*d* = +3.953
FAQ total	5.94 ± 8.58	14.68 ± 7.68	0.22 ± 0.73	<0.001	*d* = +2.975
NPI-Q total	1.93 ± 3.05	4.08 ± 3.73	0.51 ± 1.10	<0.001	*d* = +1.431
GDS total	1.18 ± 1.39	1.60 ± 1.50	0.89 ± 1.23	<0.001	*d* = +0.524
APOE ε4 carriers	140 (47.6%)	90 (76.3%)	50 (28.4%)	<0.001	*V* = 0.470
ADNI phase, 1/3	289/6	118/0	171/6	0.085	*V* = 0.118
(**b**)					
**Characteristic**	**Train**	**Validation**	**Test**	**Total**	***p***
Subjects	204	47	44	295	—
AD/CN	76/128	24/23	18/26	118/177	0.217
AD (%)	37.3	51.1	40.9	40.0	—
Slices	4080	940	880	5900	—
Age at selected scan (y)	73.65 ± 4.11	74.01 ± 4.30	72.54 ± 4.75	—	0.332
MMSE total	26.83	25.89	26.52	—	0.545
Sex, F (%)	51.0	53.2	40.9	—	0.423
APOE ε4 carriers (%)	46.8	57.4	40.9	—	0.263
Distinct sites	54	44	40	54	—

Continuous variables, Welch *t* test and Cohen’s d; categorical, Pearson χ^2^ and Cramér’s V, except ADNI phase (Fisher exact). Counts differ by variable owing to missing values: MMSE, CDR and GDS *n* = 289; FAQ and NPI-Q *n* = 268; APOE *n* = 294. Years of education and race/ethnicity are not fields in the ADNI image-collection export and were unavailable.

**Table 3:** Three-way memorisation severity classification of all 880 generated samples, graded by the nearest-neighbour distance ratio (distance to nearest training image divided by distance to nearest held-out test image) in DINOv2 feature space. A ratio below 0.5 denotes instance memorisation (near-copies of training data), a ratio from 0.5 to below 1.0 denotes distributional proximity (closer to training than to held-out data, expected for a well-trained model), and a ratio of 1.0 or above denotes distributional generalisation.

Category	Ratio Range	Count	Percentage
Instance memorisation	<0.5	0/880	0.0
Distributional proximity	0.5 to <1.0	559/880	63.5
Distributional generalisation	≥1.0	321/880	36.5

**Table 4:** Controlled preprocessing ablation. Both arms use the enhanced architecture, losses, training schedule, subject split and sampler; only the preprocessing of the input data differs. Columns report the baseline arm, the enhanced arm and the enhanced-minus-baseline difference with a 95% confidence interval, estimated by a paired subject-level bootstrap over the 44 test participants (Section 2.6.2). The difference column is the median of the paired bootstrap distribution and need not equal the subtraction of the two arm values in the preceding columns. KID and FID are lower-is-better; precision and recall are higher-is-better. The downstream AUC is a slice-level logistic-regression probe on Inception features and is not comparable with the ResNet-18 values in Section 3.5.

Measure	Baseline Preprocessing	Enhanced Preprocessing	Difference (95% CI)
KID	0.043 ± 0.004	0.028 ± 0.002	−0.0147 (−0.0231, −0.0080)
FID	50.33	46.92	−3.05 (−8.73, +2.65)
Precision	0.213	0.258	+0.069 (+0.004, +0.123)
Recall	0.380	0.385	+0.005 (−0.027, +0.043)
Downstream AUC (slice)	0.669	0.609	−0.062 (−0.140, +0.011)

**Table 5:** Contextualisation of this study against representative diffusion-based medical image synthesis work. The studies differ substantially in cohort size, image dimensionality, resolution, feature extractor, and evaluation protocol; reported values are shown as published and are not directly comparable across rows, so the table situates this study’s scale and scope rather than ranking methods. Among diffusion models applied to brain MRI, only Dhinagar et al. [13] and this study condition generation on diagnosis, and this study operates at the smallest cohort scale.

Study	Cohort	Image Data	Architecture	Diagnosis-Conditional	Reported Performance
Pinaya et al. [10]	31,740 subjects (UK Biobank)	3D T1-w, 160 × 224 × 160	LDM (VQ-VAE + diffusion)	No	Ventricular-volume conditioning *r* = 0.972
Khader et al. [12]	998 scans (ADNI subset)	3D, 64 × 64 × 64	VQ-GAN + DDPM	No	50/50 ADNI images rated realistic (radiologist)
Müller-Franzes et al. [11]	19,958–223,414 images (non-brain)	2D, 256 × 256	Medfusion (latent DDPM)	No	FID 11.63–30.03; precision 0.66–0.70
Dhinagar et al. [13]	1188 subjects/4098 scans (ADNI)	3D T1-w	Conditional LDM/DDPM	Yes (AD/CN)	+3 pp downstream AD-classification AUC
This study	295 subjects/4080 slices (ADNI)	2D coronal, 256 × 256	Class-conditional LDM (Medfusion-style)	Yes (AD/CN)	FID_∞_ 43.82; KID 0.030; TSTR AUC 0.779; 0 instance memorisation

## Data Availability

The imaging data analysed in this study were obtained from the Alzheimer’s Disease Neuroimaging Initiative (ADNI; https://adni.loni.usc.edu (accessed on 1 May 2025)) and are governed by the ADNI Data Use Agreement. They cannot be redistributed by the authors and are available to qualified researchers on application through ADNI. The analysis code is available at https://github.com/soheilfallah/brain-mri-ad-synthesis (accessed on 20 July 2026) and is publicly available. Trained model weights are not released because weights trained on ADNI images are a participant-derived artefact under the ADNI Data Use Agreement. The repository contains no ADNI participant data or participant-derived outputs, consistent with the ADNI Data Use Agreement.

## Supplementary Materials

The following supporting information can be downloaded at https://www.mdpi.com/article/10.3390/jimaging12090426/s1, Table S1: Distribution of the 295 participants across the 54 acquisition sites, and their allocation to the training, validation and test partitions. Sites are anony-mised and ordered by size rank. The median site contributes five participants, and several contribute a single diagnostic class; Table S2: Demographic, cognitive, genetic and acquisition variables by partition and diagnosis. Continuous variables are given as mean ± standard deviation with the number of non-missing observations; categorical variables as count (per cent). Cognitive instruments were not used in model training and are reported for cohort description only; Table S3: Acquisition variables by partition, with tests of association between partition and each variable. Splitting jointly on diagnosis and site controls the composition of the partitions; it does not remove scanner effects on the images themselves; Table S4: Sensitivity analysis excluding ADNI-3 participants. Effect sizes are Cohen’s d for continuous variables and Cramér’s V for categorical variables. Slice thickness is constant within the retained cohort, so no test statistic is defined; Table S5: Autoencoder reconstruction quality by coronal slice level over the full held-out test set (44 participants × 20 levels = 880 slices). Index 0 corresponds to the most anterior coordinate (MNI Y = +58 mm) and index 19 to the most posterior (MNI Y = −100 mm). Reconstruction quality is highest at the ex-treme levels, where the brain cross-section is smallest, and lowest through the mid-brain levels that carry the most anatomical detail; Table S6: Complete factor comparison of the baseline (v1) and enhanced (v2) configurations, including factors held constant. The final column records how each factor was treated in the controlled preprocessing ablation of Section 3.6: factors marked “Varies” constitute the manipulation, factors marked “Held fixed at v2” were equalised across both arms, and factors marked “Not a difference” were identical in the two configurations; Table S7: Acquisition confounding. Panel A asks whether scanner manufacturer, field strength or acquisition site can be recovered from the processed images, using subject-grouped cross-validation and balanced accuracy, for which the chance level is 1/k rather than the majority-class rate; the permutation null is 100 label shuffles. Manufacturer and site are recoverable at a small but statistically detectable margin above the permutation null; field strength is not recoverable at all. Panel B asks whether the AD versus CN signal survives when acquisition is controlled, across five designs of increasing stringency. It does: subject-level AUC ranges from 0.652 to 0.668 and every interval overlaps the reference design. With 54 sites at a median of 5.5 participants each, nine contributing a single diagnostic class, a held-out site cannot resolve an AUC difference smaller than about 0.28 (median Wilson 95% half-width at 80% accuracy), so no leave-one-site-out analysis is reported and site-blocked cross-validation (design B) is used instead; Table S8: Where the downstream classifier looks. Integrated gradients (32 steps) were computed over all 880 real held-out test slices for the classifier trained on real images and for the classifier trained on synthetic images. Panel A gives the share of total absolute attribution mass falling in each region, with 95% intervals from a 5000-replicate cluster bootstrap resampling the 44 test participants rather than the slices. The ventricular box spans MNI x from −25 to 25 mm and z from 0 to 30 mm; the hippocampal boxes span x from −38 to −18 and 18 to 38 mm with z from −30 to −8 mm; the border band is a 12-pixel ring around the brain mask boundary. Enrichment is the attribution share divided by the corresponding area share. Both classifiers place over 99% of attribution inside the brain and deplete the border band roughly 140-fold relative to its area, so neither is driven by the mask boundary or by registration residue at the brain edge, and the classifier trained on synthetic images distributes attribution almost identically to the one trained on real images. Panel B reports the integrated-gradients completeness check against a tolerance of 0.15; restricting the analysis to the images that pass leaves every value in Panel A unchanged to three decimal places; Figure S1: Sensitivity of the memorisation flagging rate to the choice of nearest-neighbour distance-ratio threshold, for the generated set and the size-matched real-image control. The dash-dotted line marks the threshold of 1.0 used in the main text. The generated and control curves are indistinguishable be-low approximately 0.90 and separate above it, reaching a maximum gap of 28.4 percentage points at a threshold of 1.02. No image in either set falls below 0.766, so no threshold at or below the instance-memorisation criterion of 0.5 flags any image; Figure S2: The eight generated images with the lowest nearest-neighbour distance ratio, shown against their nearest training neighbour. For each generated image the ratio is its distance to the nearest training image divided by its distance to the nearest held-out test image, both in DINOv2 feature space, with the training reference subsampled to 880 images so that the two reference sets are the same size. Columns are ordered by ratio, so the leftmost pair is the closest approach to the training set anywhere in the 880 generated images. Even at the minimum of 0.766 the pairs are different images at a similar anatomical level rather than near-copies: ventricular shape, sulcal pattern and brain outline all differ within every pair. No generated image fell below the 0.5 instance-memorisation threshold, and the median ratio across the full set was 0.984. The generator does not fix superior-inferior orientation, so some generated panels appear inverted relative to their neighbour; this does not affect the ratio, which is computed against two ref-erence sets that are both in the stored orientation.

## Abbreviations

The following abbreviations are used in this manuscript:

AD	Alzheimer’s Disease
ADNI	Alzheimer’s Disease Neuroimaging Initiative
ANTs	Advanced Normalisation Tools
AUC	Area Under the Receiver Operating Characteristic Curve
CDR	Clinical Dementia Rating
CI	Confidence Interval
CN	Cognitively Normal
DDIM	Denoising Diffusion Implicit Models
DDPM	Denoising Diffusion Probabilistic Models
DINOv2	Self-Distillation with No Labels, version 2
FID	Fréchet Inception Distance
GAN	Generative Adversarial Network
HD-BET	High-Definition Brain Extraction Tool
KID	Kernel Inception Distance
KL	Kullback–Leibler
LDM	Latent Diffusion Model
LPIPS	Learned Perceptual Image Patch Similarity
MNI	Montreal Neurological Institute
MRI	Magnetic Resonance Imaging
PSNR	Peak Signal-to-Noise Ratio
SSIM	Structural Similarity Index Measure
TSTR	Train on Synthetic, Test on Real
TRTR	Train on Real, Test on Real
VAE	Variational Autoencoder
VAEGAN	Variational Autoencoder with Generative Adversarial Network

## Appendix A

The baseline configuration (v1) preceded the enhanced configuration (v2) reported in the main text. The two differ in many respects at once and were never evaluated under a common harness, so no numerical comparison between them is reported (Section 2.6.1). Table A1 records the differences so that the redesign is reproducible, and Table S6 gives the complete comparison, including the factors held constant, and the scope of the confound is explicit. Input resolution is not among them; both configurations encoded 256 × 256 slices.

**Table A1:** Differences between the baseline (v1) and enhanced (v2) configurations. Autoencoder channel widths and the diffusion noise schedule are identical in the two configurations and are not listed. Parameter counts are for the trained models.

Component	Baseline (v1)	Enhanced (v2)
**Preprocessing**		
Spatial registration	None; native skull-stripped subject space	Affine, 12 DOF, to MNI152 at 182 × 218 × 182, 1 mm isotropic
Slice selection	Twenty slices linearly spaced across the central 35 to 65 per cent of each participant’s own brain extent	Twenty fixed MNI Y coordinates, +58 to −100 mm, variable density
Anatomical coverage	Central band only; 152 distinct per-participant coordinate sets	Whole brain; one coordinate set shared by all participants
Intensity normalisation	Clip at the 1st and 99th percentiles of non-zero voxels, then min–max	Clip at the 0.5th and 99.5th percentiles of brain voxels, then min–max
Resampling to 256 × 256	Centre crop or pad; no interpolation	Cubic spline interpolation from 182 × 218
Selected scan per participant	70 of 295 participants contributed a different ADNI image, 55 of them from a different visit	Reference
Subject split	Stratified on diagnosis only, 70/15/15	Stratified jointly on diagnosis and site across 54 sites
**Autoencoder**		
Latent	4 × 32 × 32	8 × 32 × 32
Parameters	54.8 M	53.8 M
Self-attention levels	Levels 3 and 4	Level 4 only
Perceptual loss	LPIPS, AlexNet backbone, weight 1.0	LPIPS, VGG backbone, weight 0.5
MS-SSIM loss	Not used	Weight 0.1
Adversarial weight	Ramped from 0 to 0.5 from epoch 10	0.01 from epoch 40
Discriminator regularisation	None	Spectral normalisation on every convolution; R1 penalty, *γ* = 10
KL schedule	Cyclical, four cycles	Monotone warm-up over 20 epochs
Checkpoint selection	Validation reconstruction plus KL	Validation reconstruction loss
Latent scale factor	1.3768	0.1852
**Diffusion model**		
U-Net channels	(128, 256, 384, 512), four levels	(128, 256, 256), three levels
Parameters	89.5 M	39.1 M
Conditioning	Additive class embedding in the timestep embedding	Cross-attention over a separate class embedding
Guidance dropout	0.2	0.15
Latents	Pre-encoded once and cached	Sampled from the posterior at every step
Training augmentation	None	Horizontal flip, *p* = 0.5
Class balancing	None	Weighted sampling
Optimiser	AdamW, 1 × 10^−4^, no schedule	AdamW, 2.5 × 10^−5^, warm-up then cosine
EMA	Not used	0.999, used for validation and all generation
**Sampling and evaluation**		
Guidance scale	4.0	2.0
DDIM steps	150	50
Generated images	900	880
Stored precision	8-bit PNG	32-bit float
FID feature extractor	TensorFlow-ported Inception	torchvision Inception v3
