# Supplementary material for: Conditional Latent Diffusion for Synthetic Brain MRI in Alzheimer’s Disease: A Preprocessing-Focused Pipeline
Source: J Imaging. 2026 Sep 9;12(9):426. doi: 10.3390/jimaging12090426 (PMC13608675; doi:10.3390/jimaging12090426)

Supplementary Materials

Conditional Latent Diffusion for Synthetic Brain MRI in Alzheimer's Disease: A Preprocessing-Focused Pipeline

Soheil Fallah and Nitsa J. Herzog *

Table S1. Distribution of the 295 participants across the 54 acquisition sites, and their allocation to the training, validation and test partitions. Sites are anonymised and ordered by size rank. The median site contributes five participants, and several contribute a single diagnostic class.

Site	n	AD	CN	Train	Val	Test		Site	n	AD	CN	Train	Val	Test
S01	15	7	8	11	2	2		S28	5	2	3	3	1	1
S02	14	7	7	10	2	2		S29	5	3	2	3	1	1
S03	13	5	8	9	2	2		S30	5	4	1	3	1	1
S04	10	5	5	7	1	2		S31	5	1	4	3	1	1
S05	9	4	5	7	1	1		S32	4	1	3	2	1	1
S06	9	3	6	7	1	1		S33	4	2	2	2	1	1
S07	8	2	6	6	1	1		S34	4	1	3	2	1	1
S08	8	5	3	6	1	1		S35	4	2	2	2	1	1
S09	8	4	4	6	1	1		S36	4	1	3	2	1	1
S10	8	4	4	6	1	1		S37	4	1	3	2	1	1
S11	8	4	4	6	1	1		S38	4	1	3	2	1	1
S12	7	3	4	5	1	1		S39	4	1	3	2	1	1
S13	7	3	4	5	1	1		S40	4	1	3	2	1	1
S14	7	3	4	5	1	1		S41	3	1	2	2	1	0
S15	7	3	4	5	1	1		S42	3	0	3	2	1	0
S16	7	4	3	5	1	1		S43	3	1	2	2	1	0
S17	7	2	5	5	1	1		S44	3	1	2	2	1	0
S18	6	3	3	4	1	1		S45	2	0	2	2	0	0
S19	6	2	4	4	1	1		S46	2	1	1	2	0	0

Site	n	AD	CN	Train	Val	Test		Site	n	AD	CN	Train	Val	Test
S20	6	3	3	4	1	1		S47	2	1	1	2	0	0
S21	6	2	4	4	1	1		S48	2	0	2	2	0	0
S22	6	1	5	4	1	1		S49	2	0	2	2	0	0
S23	6	2	4	4	1	1		S50	1	1	0	1	0	0
S24	6	2	4	4	1	1		S51	1	1	0	1	0	0
S25	6	2	4	4	1	1		S52	1	0	1	1	0	0
S26	6	2	4	4	1	1		S53	1	0	1	1	0	0
S27	6	3	3	4	1	1		S54	1	0	1	1	0	0

Table S2. Demographic, cognitive, genetic and acquisition variables by partition and diagnosis. Continuous variables are given as mean ± standard deviation with the number of non-missing observations; categorical variables as count (per cent). Cognitive instruments were not used in model training and are reported for cohort description only.

Variable	train/AD (n=76)	train/CN (n=128)	val/AD (n=24)	val/CN (n=23)	test/AD (n=18)	test/CN (n=26)
Age at selected scan (y)	72.75 ± 4.73 (n=76)	74.18 ± 3.61 (n=128)	74.48 ± 4.36 (n=24)	73.52 ± 4.28 (n=23)	70.33 ± 5.47 (n=18)	74.07 ± 3.54 (n=26)
MMSE total	23.03 ± 2.54 (n=76)	29.14 ± 0.91 (n=125)	23.00 ± 2.95 (n=24)	29.35 ± 1.14 (n=20)	22.56 ± 1.98 (n=18)	29.27 ± 1.04 (n=26)
Global CDR	0.76 ± 0.29 (n=76)	0.00 ± 0.04 (n=125)	0.77 ± 0.36 (n=24)	0.00 ± 0.00 (n=20)	0.75 ± 0.26 (n=18)	0.00 ± 0.00 (n=26)
FAQ total	13.65 ± 7.50 (n=68)	0.29 ± 0.82 (n=119)	16.05 ± 8.39 (n=21)	0.11 ± 0.46 (n=19)	17.12 ± 7.11 (n=17)	0.00 ± 0.00 (n=24)
NPI-Q total	3.78 ± 3.09 (n=68)	0.50 ± 1.10 (n=119)	5.19 ± 4.18 (n=21)	0.68 ± 1.34 (n=19)	3.94 ± 5.24 (n=17)	0.42 ± 0.93 (n=24)
GDS total	1.57 ± 1.58 (n=76)	0.98 ± 1.35 (n=125)	1.46 ± 1.28 (n=24)	0.80 ± 0.83 (n=20)	1.94 ± 1.47 (n=18)	0.54 ± 0.76 (n=26)
Sex	F: 41 (53.9%); M: 35 (46.1%) [n=76]	F: 63 (49.2%); M: 65 (50.8%) [n=128]	F: 12 (50.0%); M: 12 (50.0%) [n=24]	F: 13 (56.5%); M: 10 (43.5%) [n=23]	F: 5 (27.8%); M: 13 (72.2%) [n=18]	F: 13 (50.0%); M: 13 (50.0%) [n=26]
APOE4 carriage	Non-carrier: 18 (23.7%); Carrier: 58 (76.3%) [n=76]	Non-carrier: 90 (70.9%); Carrier: 37 (29.1%) [n=127]	Non-carrier: 4 (16.7%); Carrier: 20 (83.3%) [n=24]	Non-carrier: 16 (69.6%); Carrier: 7 (30.4%) [n=23]	Non-carrier: 6 (33.3%); Carrier: 12 (66.7%) [n=18]	Non-carrier: 20 (76.9%); Carrier: 6 (23.1%) [n=26]

Variable	train/AD (n=76)	train/CN (n=128)	val/AD (n=24)	val/CN (n=23)	test/AD (n=18)	test/CN (n=26)
APOE genotype	2/4: 3 (3.9%); 3/3: 18 (23.7%); 3/4: 35 (46.1%); 4/4: 20 (26.3%) [n=76]	2/2: 1 (0.8%); 2/3: 17 (13.4%); 2/4: 1 (0.8%); 3/3: 72 (56.7%); 3/4: 32 (25.2%); 4/4: 4 (3.1%) [n=127]	2/3: 2 (8.3%); 3/3: 2 (8.3%); 3/4: 15 (62.5%); 4/4: 5 (20.8%) [n=24]	2/3: 2 (8.7%); 2/4: 2 (8.7%); 3/3: 14 (60.9%); 3/4: 3 (13.0%); 4/4: 2 (8.7%) [n=23]	2/3: 1 (5.6%); 3/3: 5 (27.8%); 3/4: 10 (55.6%); 4/4: 2 (11.1%) [n=18]	2/2: 1 (3.8%); 2/3: 4 (15.4%); 3/3: 15 (57.7%); 3/4: 6 (23.1%) [n=26]
ADNI phase at scan	ADNI 1: 76 (100.0%) [n=76]	ADNI 1: 125 (97.7%); ADNI 3: 3 (2.3%) [n=128]	ADNI 1: 24 (100.0%) [n=24]	ADNI 1: 20 (87.0%); ADNI 3: 3 (13.0%) [n=23]	ADNI 1: 18 (100.0%) [n=18]	ADNI 1: 26 (100.0%) [n=26]
Global CDR (categorical)	CDR 0.5: 38 (50.0%); CDR 1: 37 (48.7%); CDR 2: 1 (1.3%) [n=76]	CDR 0: 124 (99.2%); CDR 0.5: 1 (0.8%) [n=125]	CDR 0.5: 13 (54.2%); CDR 1: 10 (41.7%); CDR 2: 1 (4.2%) [n=24]	CDR 0: 20 (100.0%) [n=20]	CDR 0.5: 9 (50.0%); CDR 1: 9 (50.0%) [n=18]	CDR 0: 26 (100.0%) [n=26]
Scanner manufacturer	GE MEDICAL SYSTEMS: 35 (46.1%); Philips Medical Systems: 5 (6.6%); SIEMENS: 36 (47.4%) [n=76]	GE MEDICAL SYSTEMS: 57 (44.5%); Philips Medical Systems: 13 (10.2%); SIEMENS: 58 (45.3%) [n=128]	GE MEDICAL SYSTEMS: 12 (50.0%); Philips Medical Systems: 2 (8.3%); SIEMENS: 10 (41.7%) [n=24]	GE MEDICAL SYSTEMS: 8 (34.8%); SIEMENS: 15 (65.2%) [n=23]	GE MEDICAL SYSTEMS: 10 (55.6%); Philips Medical Systems: 1 (5.6%); SIEMENS: 7 (38.9%) [n=18]	GE MEDICAL SYSTEMS: 12 (46.2%); Philips Medical Systems: 2 (7.7%); SIEMENS: 12 (46.2%) [n=26]
Scanner model	Achieva: 1 (1.3%); Avanto: 6 (7.9%); GENESIS_SIGNA: 5 (6.6%); Gyroscan Intera: 3 (3.9%); Intera: 1 (1.3%); SIGNA EXCITE: 24 (31.6%); SIGNA HDx: 6 (7.9%); Sonata: 9 (11.8%); SonataVision: 1 (1.3%); Symphony: 13 (17.1%); Trio: 4 (5.3%);	Achieva: 1 (0.8%); Achieva dStream: 1 (0.8%); Avanto: 16 (12.5%); GENESIS_SIGNA: 7 (5.5%); Gyroscan Intera: 4 (3.1%); Intera: 7 (5.5%); Prisma: 1 (0.8%); Prisma_fit: 1 (0.8%); SIGNA EXCITE: 48 (37.5%); SIGNA HDx: 2 (1.6%);	Avanto: 3 (12.5%); Gyroscan Intera: 1 (4.2%); Intera: 1 (4.2%); SIGNA EXCITE: 10 (41.7%); SIGNA HDx: 2 (8.3%); Sonata: 3 (12.5%); Symphony: 3 (12.5%); TrioTim: 1 (4.2%) [n=24]	Avanto: 5 (21.7%); GENESIS_SIGNA: 1 (4.3%); Prisma: 1 (4.3%); Prisma_fit: 2 (8.7%); SIGNA EXCITE: 7 (30.4%); Sonata: 2 (8.7%); Symphony: 5 (21.7%) [n=23]	Achieva: 1 (5.6%); Avanto: 4 (22.2%); GENESIS_SIGNA: 2 (11.1%); SIGNA EXCITE: 6 (33.3%); SIGNA HDx: 2 (11.1%); Symphony: 3 (16.7%) [n=18]	Allegra: 1 (3.8%); Avanto: 1 (3.8%); GENESIS_SIGNA: 1 (3.8%); Intera: 2 (7.7%); SIGNA EXCITE: 10 (38.5%); SIGNA HDx: 1 (3.8%); Sonata: 4 (15.4%); Symphony: 4 (15.4%); SymphonyTim: 1 (3.8%); Trio: 1 (3.8%) [n=26]

Variable	train/AD (n=76)	train/CN (n=128)	val/AD (n=24)	val/CN (n=23)	test/AD (n=18)	test/CN (n=26)
	TrioTim: 3 (3.9%) [n=76]	Sonata: 13 (10.2%); SonataVision: 1 (0.8%); Symphony: 23 (18.0%); Trio: 3 (2.3%) [n=128]				
Field strength (T)	1.5: 66 (86.8%); 2.9: 4 (5.3%); 3.0: 6 (7.9%) [n=76]	1.5: 116 (90.6%); 2.9: 3 (2.3%); 3.0: 9 (7.0%) [n=128]	1.5: 23 (95.8%); 3.0: 1 (4.2%) [n=24]	1.5: 20 (87.0%); 3.0: 3 (13.0%) [n=23]	1.5: 17 (94.4%); 3.0: 1 (5.6%) [n=18]	1.5: 24 (92.3%); 2.9: 2 (7.7%) [n=26]
Acquisition type	3D: 74 (100.0%) [n=74]	3D: 124 (100.0%) [n=124]	3D: 23 (100.0%) [n=23]	3D: 23 (100.0%) [n=23]	3D: 17 (100.0%) [n=17]	3D: 25 (100.0%) [n=25]
Acquisition plane	SAGITTAL: 76 (100.0%) [n=76]	SAGITTAL: 128 (100.0%) [n=128]	SAGITTAL: 24 (100.0%) [n=24]	SAGITTAL: 23 (100.0%) [n=23]	SAGITTAL: 18 (100.0%) [n=18]	SAGITTAL: 26 (100.0%) [n=26]
Weighting	T1: 76 (100.0%) [n=76]	T1: 128 (100.0%) [n=128]	T1: 24 (100.0%) [n=24]	T1: 23 (100.0%) [n=23]	T1: 18 (100.0%) [n=18]	T1: 26 (100.0%) [n=26]
Distinct sites	36	51	24	22	18	23

Table S3. Acquisition variables by partition, with tests of association between partition and each variable. Splitting jointly on diagnosis and site controls the composition of the partitions; it does not remove scanner effects on the images themselves.

Acquisition variable	Train (n=204)	Validation (n=47)	Test (n=44)	Test across splits	Statistic	df	p	Effect size
Scanner manufacturer	GE MEDICAL SYSTEMS: 92 (45.1%); Philips Medical Systems: 18 (8.8%); SIEMENS: 94 (46.1%) [n=204]	GE MEDICAL SYSTEMS: 20 (42.6%); Philips Medical Systems: 2 (4.3%); SIEMENS: 25 (53.2%) [n=47]	GE MEDICAL SYSTEMS: 22 (50.0%); Philips Medical Systems: 3 (6.8%); SIEMENS: 19 (43.2%) [n=44]	Pearson chi-square	chi2=1.935 (min exp=3.43)	4	0.748	V=0.057

Acquisition variable	Train (n=204)	Validation (n=47)	Test (n=44)	Test across splits	Statistic	df	p	Effect size
Scanner model	Achieva: 2 (1.0%); Achieva dStream: 1 (0.5%); Avanto: 22 (10.8%); GENESIS_SIGNA: 12 (5.9%); Gyroscan Intera: 7 (3.4%); Intera: 8 (3.9%); Prisma: 1 (0.5%); Prisma_fit: 1 (0.5%); SIGNA EXCITE: 72 (35.3%); SIGNA HDx: 8 (3.9%); Sonata: 22 (10.8%); SonataVision: 2 (1.0%); Symphony: 36 (17.6%); Trio: 7 (3.4%); TrioTim: 3 (1.5%) [n=204]	Avanto: 8 (17.0%); GENESIS_SIGNA: 1 (2.1%); Gyroscan Intera: 1 (2.1%); Intera: 1 (2.1%); Prisma: 1 (2.1%); Prisma_fit: 2 (4.3%); SIGNA EXCITE: 17 (36.2%); SIGNA HDx: 2 (4.3%); Sonata: 5 (10.6%); Symphony: 8 (17.0%); TrioTim: 1 (2.1%) [n=47]	Achieva: 1 (2.3%); Allegra: 1 (2.3%); Avanto: 5 (11.4%); GENESIS_SIGNA: 3 (6.8%); Intera: 2 (4.5%); SIGNA EXCITE: 16 (36.4%); SIGNA HDx: 3 (6.8%); Sonata: 4 (9.1%); Symphony: 7 (15.9%); SymphonyTim: 1 (2.3%); Trio: 1 (2.3%) [n=44]	Pearson chi-square	chi2=29.520 (min exp=0.15)	32	0.593	V=0.224
Field strength (T)	1.5: 182 (89.2%); 2.9: 7 (3.4%); 3.0: 15 (7.4%) [n=204]	1.5: 43 (91.5%); 3.0: 4 (8.5%) [n=47]	1.5: 41 (93.2%); 2.9: 2 (4.5%); 3.0: 1 (2.3%) [n=44]	Pearson chi-square	chi2=3.552 (min exp=1.34)	4	0.47	V=0.078
Field strength collapsed	1.5 T: 182 (89.2%); 3 T (incl. 2.9): 22 (10.8%) [n=204]	1.5 T: 43 (91.5%); 3 T (incl. 2.9): 4 (8.5%) [n=47]	1.5 T: 41 (93.2%); 3 T (incl. 2.9): 3 (6.8%) [n=44]	Pearson chi-square	chi2=0.752 (min exp=4.33)	2	0.687	V=0.050
Acquisition type	3D: 198 (100.0%) [n=198]	3D: 46 (100.0%) [n=46]	3D: 42 (100.0%) [n=42]	not testable (constant: 3D)	-	-	-	-
Acquisition plane	SAGITTAL: 204 (100.0%) [n=204]	SAGITTAL: 47 (100.0%) [n=47]	SAGITTAL: 44 (100.0%) [n=44]	not testable (constant: SAGITTAL)	-	-	-	-
Weighting	T1: 204 (100.0%) [n=204]	T1: 47 (100.0%) [n=47]	T1: 44 (100.0%) [n=44]	not testable (constant: T1)	-	-	-	-

Acquisition variable	Train (n=204)	Validation (n=47)	Test (n=44)	Test across splits	Statistic	df	p	Effect size
Slice thickness (mm)	1.20 ± 0.02 (n=204)	1.19 ± 0.05 (n=47)	1.20 ± 0.00 (n=44)	-	-	-	-	-
Matrix Z	165.89 ± 8.33 (n=204)	171.06 ± 27.01 (n=47)	165.45 ± 7.78 (n=44)	-	-	-	-	-
ADNI phase at scan	ADNI 1: 201 (98.5%); ADNI 3: 3 (1.5%) [n=204]	ADNI 1: 44 (93.6%); ADNI 3: 3 (6.4%) [n=47]	ADNI 1: 44 (100.0%) [n=44]	Pearson chi-square	chi2=5.700 (min exp=0.89)	2	0.0578	V=0.139

Table S4. Sensitivity analysis excluding ADNI-3 participants. Effect sizes are Cohen's d for continuous variables and Cramér's V for categorical variables. Slice thickness is constant within the retained cohort, so no test statistic is defined.

Variable	Values after excluding ADNI-3	Statistic	p (Welch / MWU)	Effect size
Age at selected scan (y)	AD 72.73 ± 4.89 (n=118) vs CN 74.26 ± 3.55 (n=171)	t=-2.901	0.00413 / 0.00944	d=-0.368
Slice thickness (mm)	AD 1.20 ± 0.00 (n=118) vs CN 1.20 ± 0.00 (n=171)	t=nan	n/a / n/a	d=+nan
MMSE total	AD 22.95 ± 2.54 (n=118) vs CN 29.19 ± 0.96 (n=171)	t=-25.475	1.84e-54 / 2.63e-48	d=-3.504
Field strength (T)	1.5: 266 (92.0%); 2.9: 9 (3.1%); 3.0: 14 (4.8%) [n=289]	chi2=1.697	0.428	V=0.077
ADNI phase at scan	constant after exclusion	-	-	-

Table S5. Autoencoder reconstruction quality by coronal slice level over the full held-out test set (44 participants × 20 levels = 880 slices). Index 0 corresponds to the most anterior coordinate (MNI Y = +58 mm) and index 19 to the most posterior (MNI Y = −100 mm). Reconstruction quality is highest at the extreme levels, where the brain cross-section is smallest, and lowest through the mid-brain levels that carry the most anatomical detail.

Coronal slice index	n	PSNR (dB), mean ± SD	SSIM, mean ± SD
0	44	41.82 ± 2.14	0.9975 ± 0.0005
1	44	41.23 ± 2.35	0.9948 ± 0.0012
2	44	39.22 ± 1.73	0.9928 ± 0.0013
3	44	38.73 ± 1.73	0.9898 ± 0.0017
4	44	38.87 ± 1.04	0.9878 ± 0.0022
5	44	38.56 ± 1.17	0.9867 ± 0.0022
6	44	38.22 ± 1.22	0.9865 ± 0.0021
7	44	38.31 ± 0.92	0.9863 ± 0.0023
8	44	38.23 ± 1.07	0.9860 ± 0.0024
9	44	38.36 ± 1.10	0.9861 ± 0.0023
10	44	38.15 ± 1.03	0.9862 ± 0.0022
11	44	37.62 ± 0.90	0.9844 ± 0.0025
12	44	37.18 ± 1.04	0.9839 ± 0.0025
13	44	37.25 ± 0.95	0.9834 ± 0.0028
14	44	37.35 ± 0.89	0.9840 ± 0.0027
15	44	37.39 ± 1.07	0.9857 ± 0.0028
16	44	37.59 ± 1.91	0.9884 ± 0.0026
17	44	37.99 ± 2.79	0.9912 ± 0.0021
18	44	39.54 ± 2.75	0.9947 ± 0.0015
19	44	41.60 ± 3.49	0.9976 ± 0.0009

Table S6. Complete factor comparison of the baseline (v1) and enhanced (v2) configurations, including factors held constant. The final column records how each factor was treated in the controlled preprocessing ablation of Section 3.6: factors marked "Varies" constitute the manipulation, factors marked "Held fixed at v2" were equalised across both arms, and factors marked "Not a difference" were identical in the two configurations.

**Stage**	**Factor**	**Baseline (v1)**	**Enhanced (v2)**	**Status in the ablation**
Preprocessing	Registration	None; native skull-stripped subject space	Affine, 12 DOF, to MNI152 at 182 × 218 × 182, 1 mm	Varies
	Slice selection	Twenty slices, linearly spaced over the central 35–65% of each participant's brain extent	Twenty fixed MNI Y coordinates, +58 to −100 mm	Varies
	Anatomical coverage	Central band; 152 distinct per-participant coordinate sets	Whole brain; one shared coordinate set	Varies
	Intensity normalisation	Clip 1st/99th percentile of non-zero voxels, then min–max	Clip 0.5th/99.5th percentile of brain voxels, then min–max	Varies
	Resampling to 256 × 256	Centre crop or pad; no interpolation	Cubic spline interpolation from 182 × 218	Varies
	Selected scan per participant	70 of 295 participants contributed a different ADNI image; 55 from a different visit	Reference	Residual confound
	Subject split	Stratified on diagnosis only, 70/15/15	Stratified on diagnosis and site, 54 sites	Held fixed at v2
Autoencoder	Input dynamic range	[−1, 1]	[0, 1]	Held fixed at v2
	Latent channels	4 (4 × 32 × 32)	8 (8 × 32 × 32)	Held fixed at v2
	Channel widths	(64, 128, 256, 512)	(64, 128, 256, 512)	Identical
	Self-attention levels	Levels 3 and 4	Level 4 only	Held fixed at v2
	Parameters	54,824,961	53,807,265	Held fixed at v2
	Perceptual loss	LPIPS, AlexNet backbone, weight 1.0	LPIPS, VGG backbone, weight 0.5	Held fixed at v2
	MS-SSIM loss	Not used	Weight 0.1	Held fixed at v2
	Adversarial weight and onset	Ramp 0 → 0.5 from epoch 10 over 20 epochs	0.01 from epoch 40	Held fixed at v2
	Adversarial formulation	Hand-written hinge (D); non-saturating (G)	PatchAdversarialLoss (hinge), both sides	Held fixed at v2

**Stage**	**Factor**	**Baseline (v1)**	**Enhanced (v2)**	**Status in the ablation**
	Discriminator regularisation	BatchNorm PatchGAN; no spectral norm, no R1	Spectral norm on every convolution; R1, $\gamma = 10$	Held fixed at v2
	Discriminator-input augmentation	None	Horizontal flip 0.5; ColorJitter (0.05, 0.05); $N(0, 0.02^2)$	Held fixed at v2
	KL schedule	Cyclical, four cycles, max 1×10^{-6}	Monotone min(epoch/20, 1) × 1×10^{-6}	Held fixed at v2
	Epoch budget	200, run to completion; best epoch 151	Max 300, patience 30; ran 100, best 70	Held fixed at v2
	Checkpoint selection	Validation L1 reconstruction + KL	Validation L1 reconstruction	Held fixed at v2
	Optimiser	AdamW, 1×10^{-4} (AE) / 5×10^{-5} (D), wd 0.01	AdamW, 1×10^{-4}, wd 1×10^{-4}	Held fixed at v2
	Latent scale factor	1.3768	0.1852	Recomputed per arm
Diffusion model	U-Net channels and levels	(128, 256, 384, 512), four levels	(128, 256, 256), three levels	Held fixed at v2
	Attention levels	(F, T, T, T)	(F, T, T)	Held fixed at v2
	Parameters	89,539,460	39,105,544	Held fixed at v2
	Conditioning	Additive class embedding in the timestep embedding, (3, 512)	Cross-attention over nn.Embedding(3, 64)	Held fixed at v2
	Unconditional token	Class index 2 inside the U-Net	Label −1 remapped to index 2	Held fixed at v2
	Guidance label dropout	0.2	0.15	Held fixed at v2
	Latent supply	Pre-encoded once and cached; frozen	Encoded on the fly from the posterior	Held fixed at v2
	Training augmentation	None	Horizontal flip, $p \approx 0.5$	Held fixed at v2
	Class balancing	None (plain shuffle)	WeightedRandomSampler	Held fixed at v2
	Batch size	32	16	Held fixed at v2
	Learning rate and schedule	1×10^{-4} AdamW, no schedule	2.5×10^{-5}, 500-step warm-up, cosine to 1×10^{-6}	Held fixed at v2
	EMA	Not used	0.999 on U-Net and class embedding	Held fixed at v2
	Epoch budget	300 configured, 116 run, best 23	150, patience 25; ran 121, best 95	Held fixed at v2

Stage	Factor	Baseline (v1)	Enhanced (v2)	Status in the ablation
	Noise schedule	scaled_linear_beta, 0.0015 → 0.0195, T = 1000	Identical	Identical
Sampling and evaluation	Guidance scale	4.0	2.0	Held fixed at v2
	DDIM steps	150	50	Held fixed at v2
	Generated images	900 (540 CN, 360 AD)	880 (520 CN, 360 AD)	Held fixed at 880
	Stored precision	8-bit PNG (quantised)	32-bit float	Held fixed at float32
	FID feature extractor	torchmetrics (TensorFlow-ported Inception)	torchvision Inception v3	Held fixed at torchvision
	Input resolution	256 × 256	256 × 256	Not a difference
	Cohort	Same 295 participants	Same 295 participants	Not a difference

Table S7. Acquisition confounding. Panel A asks whether scanner manufacturer, field strength or acquisition site can be recovered from the processed images, using subject-grouped cross-validation and balanced accuracy, for which the chance level is 1/k rather than the majority-class rate; the permutation null is 100 label shuffles. Manufacturer and site are recoverable at a small but statistically detectable margin above the permutation null; field strength is not recoverable at all. Panel B asks whether the AD versus CN signal survives when acquisition is controlled, across five designs of increasing stringency. It does: subject-level AUC ranges from 0.652 to 0.668 and every interval overlaps the reference design. With 54 sites at a median of 5.5 participants each, nine contributing a single diagnostic class, a held-out site cannot resolve an AUC difference smaller than about 0.28 (median Wilson 95% half-width at 80% accuracy), so no leave-one-site-out analysis is reported and site-blocked cross-validation (design B) is used instead.

Panel A. Prediction of acquisition metadata from the processed real images.

Target	k	Chance (1/k)	Balanced accuracy, slice (95% CI)	Subject	Permutation null	p
Scanner manufacturer, logistic regression	3	0.333	0.350 (0.338 to 0.363)	0.364	0.323 ± 0.012	0.010
Scanner manufacturer, random forest	3	0.333	0.374 (0.360 to 0.388)	0.443	0.330 ± 0.007	0.048
Field strength, 1.5 T vs 3 T, logistic regression	2	0.500	0.500 (0.498 to 0.502)	0.500	0.500 ± 0.002	0.396
Field strength, random forest	2	0.500	0.499 (0.498 to 0.500)	0.500	0.500 ± 0.001	0.762

Target	k	Chance (1/k)	Balanced accuracy, slice (95% CI)	Subject	Permutation null	p
Acquisition site, 27 sites with at least 5 participants	27	0.037	0.045 (0.037 to 0.052)	0.055	0.030 ± 0.004	0.039

Panel B. AD versus CN under acquisition control.

Design	What it controls	n	Subject AUC (95% CI)
A. Subject-grouped 5-fold	Participant leakage only (reference)	295	0.668 (0.605 to 0.728)
B. Site-blocked 5-fold	No site on both sides of a fold boundary	295	0.662 (0.598 to 0.723)
C. Leave-one-manufacturer-out	Held-out vendor never seen in training	295	0.653 (0.586 to 0.714)
D. 1.5 T only	Field strength held constant	266	0.652 (0.581 to 0.714)
E. Manufacturer-matched 1:1, 20 draws	AD and CN matched within manufacturer	236	0.660 (0.613 to 0.697)

Table S8. Where the downstream classifier looks. Integrated gradients (32 steps) were computed over all 880 real held-out test slices for the classifier trained on real images and for the classifier trained on synthetic images. Panel A gives the share of total absolute attribution mass falling in each region, with 95% intervals from a 5,000-replicate cluster bootstrap resampling the 44 test participants rather than the slices. The ventricular box spans MNI x from −25 to 25 mm and z from 0 to 30 mm; the hippocampal boxes span x from −38 to −18 and 18 to 38 mm with z from −30 to −8 mm; the border band is a 12-pixel ring around the brain mask boundary. Enrichment is the attribution share divided by the corresponding area share. Both classifiers place over 99% of attribution inside the brain and deplete the border band roughly 140-fold relative to its area, so neither is driven by the mask boundary or by registration residue at the brain edge, and the classifier trained on synthetic images distributes attribution almost identically to the one trained on real images. Panel B reports the integrated-gradients completeness check against a tolerance of 0.15; restricting the analysis to the images that pass leaves every value in Panel A unchanged to three decimal places.

Panel A. Share of total attribution mass by region.

Region	Share of frame	Trained on real	Trained on synthetic
Inside brain mask	0.374	0.9911 (0.9905 to 0.9915)	0.9915 (0.9910 to 0.9919)
Outside brain mask	0.626	0.0089 (0.0085 to 0.0095)	0.0085 (0.0081 to 0.0090)
Border band, 12 px	0.179	0.0011 (0.0009 to 0.0014)	0.0013 (0.0011 to 0.0016)
Ventricular box	—	0.1347 (0.1313 to 0.1381)	0.1433 (0.1400 to 0.1465)

Region	Share of frame	Trained on real	Trained on synthetic
Hippocampal boxes	—	0.0776 (0.0756 to 0.0795)	0.0728 (0.0710 to 0.0746)

Enrichment (attribution share divided by area share).

	Trained on real	Trained on synthetic
Brain	2.650	2.651
Border band	0.0063	0.0073

Panel B. Completeness of the attributions.

	Trained on real	Trained on synthetic
Median relative error	0.062	0.114
Mean relative error	0.249	0.493
Images within tolerance	75.5%	57.7%

Figure S1. Sensitivity of the memorisation flagging rate to the choice of nearest-neighbour distance-ratio threshold, for the generated set and the size-matched real-image control. The dash-dotted line marks the threshold of 1.0 used in the main text. The generated and control curves are indistinguishable below approximately 0.90 and separate above it, reaching a maximum gap of 28.4 percentage points at a threshold of 1.02. No image in either set falls below 0.766, so no threshold at or below the instance-memorisation criterion of 0.5 flags any image.

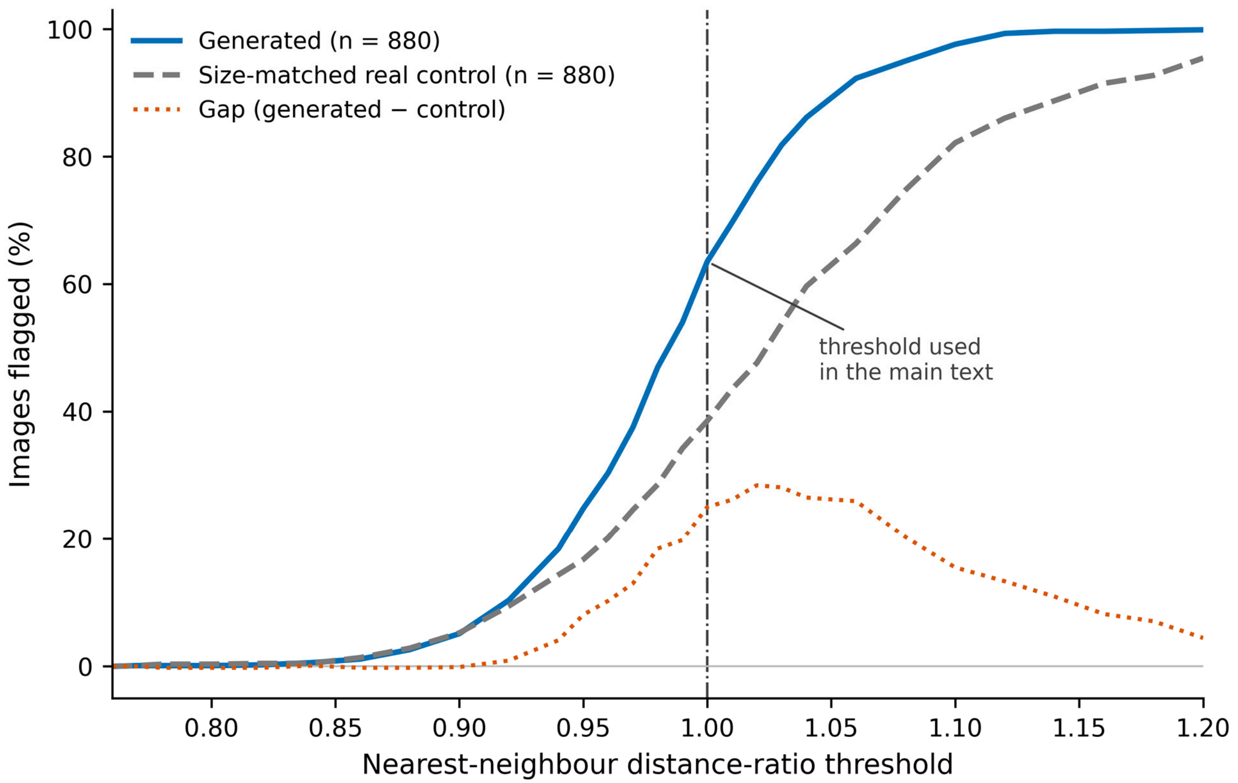

Figure S2. The eight generated images with the lowest nearest-neighbour distance ratio, shown against their nearest training neighbour. For each generated image the ratio is its distance to the nearest training image divided by its distance to the nearest held-out test image, both in DINOv2 feature space, with the training reference subsampled to 880 images so that the two reference sets are the same size. Columns are ordered by ratio, so the leftmost pair is the closest approach to the training set anywhere in the 880 generated images. Even at the minimum of 0.766 the pairs are different images at a similar anatomical level rather than near-copies: ventricular shape, sulcal pattern and brain outline all differ within every pair. No generated image fell below the 0.5 instance-memorisation threshold, and the median ratio across the full set was 0.984. The generator does not fix superior-inferior orientation, so some generated panels appear inverted relative to their neighbour; this does not affect the ratio, which is computed against two reference sets that are both in the stored orientation.

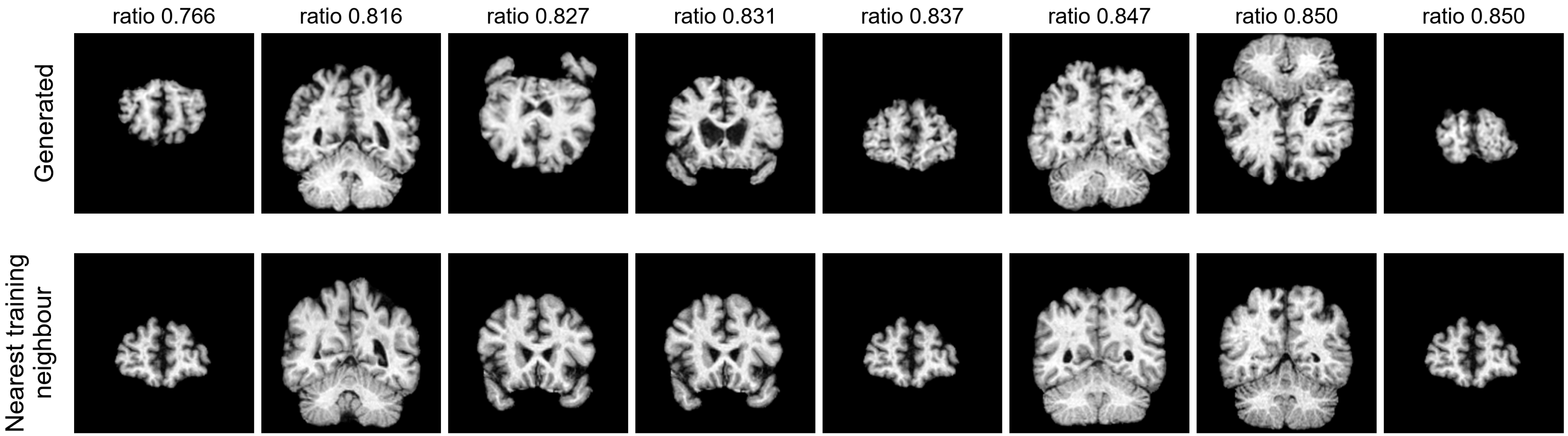

Supplement: Supplementary file 1 [file jimaging-12-00426-s001.zip › jimaging-4478369-supplementary.pdf]
